# Ethanol yield improvement in *Saccharomyces cerevisiae GPD2 Delta FPS1 Delta ADH2 Delta DLD3 Delta* mutant and molecular mechanism exploration based on the metabolic flux and transcriptomics approaches

**DOI:** 10.1186/s12934-022-01885-3

**Published:** 2022-08-13

**Authors:** Peizhou Yang, Shuying Jiang, Shuhua Lu, Suwei Jiang, Shaotong Jiang, Yanhong Deng, Jiuling Lu, Hu Wang, Yong Zhou

**Affiliations:** 1 College of Food and Biological Engineering, Anhui Key Laboratory of Intensive Processing of Agricultural Products, Hefei University of Technology, 420 Feicui Road, Shushan District, Hefei, 230601 Anhui China; 2 Department of Biological, Food and Environment Engineering, Hefei University, 158 Jinxiu Avenue, Hefei, 230601 China; 3 Suzhou Cofco Biochemical Co., Ltd., Suzhou, 234001 China

**Keywords:** Engineered *Saccharomyces cerevisiae*, Ethanol production, Gene knock-out, CRISPR-Cas9, Transcriptomics analysis, Metabolic flux

## Abstract

**Background:**

*Saccharomyces cerevisiae* generally consumes glucose to produce ethanol accompanied by the main by-products of glycerol, acetic acid, and lactic acid. The minimization of the formation of by-products in *S. cerevisiae* was an effective way to improve the economic viability of the bioethanol industry. In this study, *S. cerevisiae GPD2*, *FPS1*, *ADH2*, and *DLD3* genes were knocked out by the Clustered Regularly Interspaced Short Palindromic Repeats Cas9 (CRISPR-Cas9) approach. The mechanism of gene deletion affecting ethanol metabolism was further elucidated based on metabolic flux and transcriptomics approaches.

**Results:**

The engineered *S. cerevisiae* with gene deletion of *GPD2*, *FPS1*, *ADH2*, and *DLD3* was constructed by the CRISPR-Cas9 approach. The ethanol content of engineered *S. cerevisiae GPD2 Delta FPS1 Delta ADH2 Delta DLD3 Delta* increased by 18.58% with the decrease of glycerol, acetic acid, and lactic acid contents by 22.32, 8.87, and 16.82%, respectively. The metabolic flux analysis indicated that the carbon flux r_ethanol_ in engineered strain increased from 60.969 to 63.379. The sequencing-based RNA-Seq transcriptomics represented 472 differential expression genes (DEGs) were identified in engineered *S. cerevisiae*, in which 195 and 277 genes were significantly up-regulated and down-regulated, respectively. The enriched pathways of up-regulated genes were mainly involved in the energy metabolism of carbohydrates, while the down-regulated genes were mainly enriched in acid metabolic pathways.

**Conclusions:**

The yield of ethanol in engineered *S. cerevisiae* increased with the decrease of the by-products including glycerol, acetic acid, and lactic acid. The deletion of genes *GPD2*, *FPS1*, *ADH2*, and *DLD3* resulted in the redirection of carbon flux.

**Supplementary Information:**

The online version contains supplementary material available at 10.1186/s12934-022-01885-3.

## Background

Ethanol mainly produced by *Saccharomyces cerevisiae* is widely applied in the chemical industry, beverages, bioethanol, pharmaceuticals, and cosmetics [1]. 90–95% of ethanol is produced via the anaerobic fermentation approach [2]. Ethanol fermentation biochemistry includes substrate degradation pathways (glycolysis, alcoholic and glyceropyruvic fermentation, xylose catabolic pathways, and glycerol assimilation) and metabolic regulation pathways between fermentation and respiration (Pasteur effect, Kluyver effect, Crabtree effect, and Custers effect) [3–6]. In the process of carbon flow metabolism in *S. cerevisiae*, 5% of carbon source is converted into glycerol as a byproduct [7]. Although glycerol plays a physiological role in osmoregulation and regulating redox balance, the excessive formation of glycerol will reduce the utilization rate of sugar, and then affect the production rate of ethanol [8].

The glycerol formation can be effectively regulated by knocking out the glycerol formation pathway, preventing glycerol secretion, and regulating redox balance. Therefore, the redirection of carbon flow and the influence of the intracellular redox potential pathway will affect glycerol formation and ethanol production. Glycerol is produced from dihydroxyacetone phosphate (DHAP) in the presence of glycerol-3-phosphate dehydrogenase and glycerol-3-phosphatase phosphatase. NAD+ dependent glycerol-3-phosphate dehydrogenase has two isoforms of Gpd1 and Gpd2 in *S. cerevisiae*, which catalyze the reduction of DHAP to glycerol-3-phosphate by synergetic catalysis approach [9, 10]. Redox cofactors play a role in cellular metabolism by participating in numerous biochemical reactions [11]. The maintenance of redox balance is a fundamental requirement for cellular metabolism and cell growth [12]. Thus, *GPD2* affects the carbon metabolic pathway of glycerol formation by regulating redox potential.

In *S. cerevisiae*, ethanol is produced from pyruvate via the following two steps: decarboxylation of pyruvate to acetaldehyde by pyruvate decarboxylase; acetaldehyde is further reduced to ethanol by alcohol dehydrogenase (Adh). *ADH2* encoding for alcohol dehydrogenase II catalyzes ethanol oxidation toward acetaldehyde [13]. Thus, during the pathway engineering, less ethanol is consumed by the disruption of alcohol dehydrogenase 2 (Adh2) activity [14]. In addition, *ADH2* deletion is involved in the increased demand for NAD^+^ regeneration in the carbon metabolism. Both cofactor removal and consumption reduction could result in the metabolic cofactor imbalance [15, 16]. Glycerol is mainly exported across the plasma membrane in *S. cerevisiae* through the protein channel Fps1 regulated by extracellular osmolarity [17, 18]. Fps1 is a member of the major intrinsic protein (MIP) family as channel proteins, which contain six transmembrane domains [19]. *S. cerevisiae* fps1Δ mutant exhibits the intracellular accumulation of glycerol. The glycerol accumulation triggers other regulatory systems for the reduction of glycerol biosynthesis, which then results in an increase of ethanol yield [20]. In addition, d-lactate dehydrogenase 3 (Dld3) in *S. cerevisiae* is involved in the conversion of d-lactate to pyruvate [21].

**Fig. 1:**
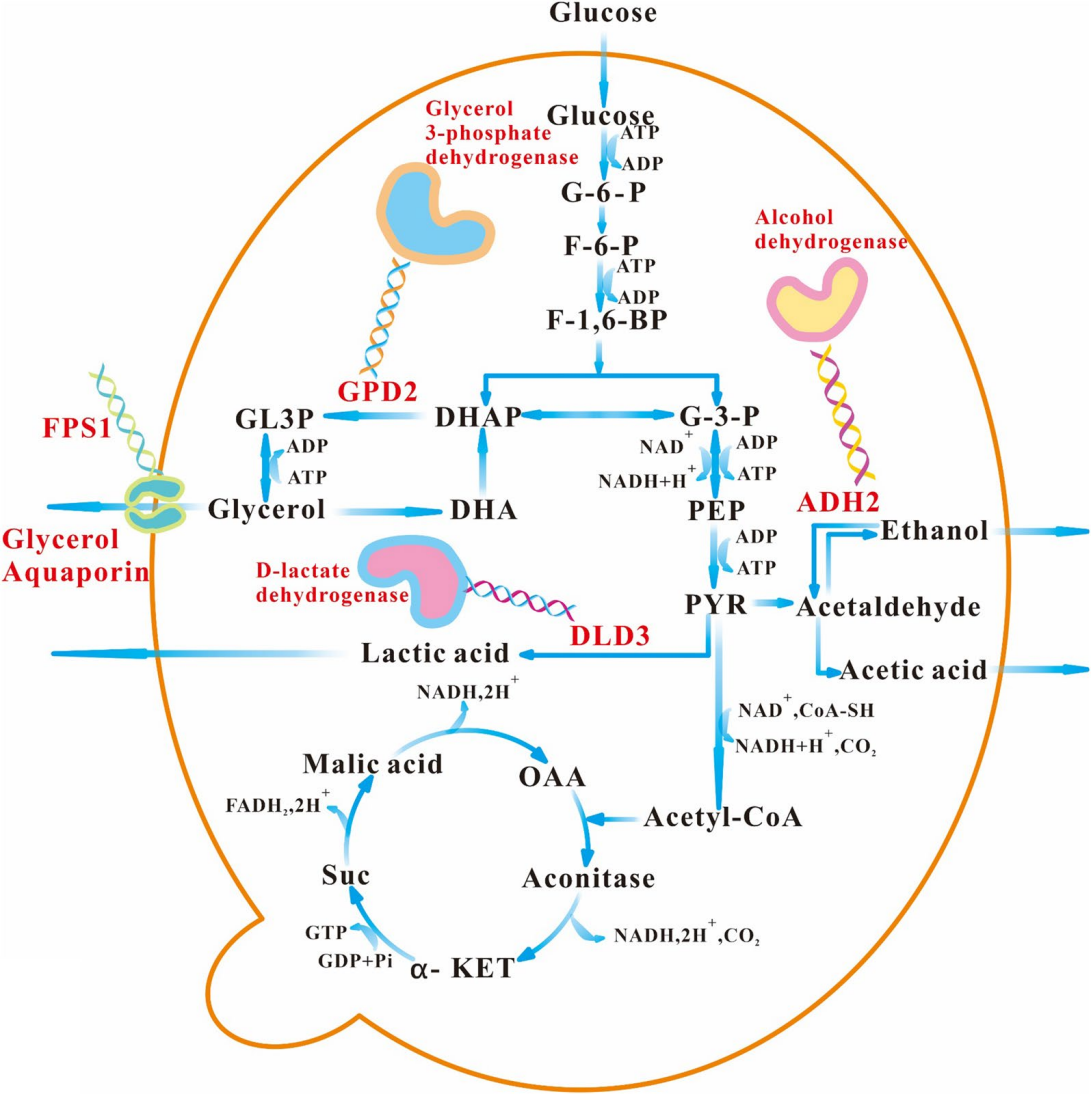
Modification of carbon metabolism pathway of *S. cerevisiae* for improvement of ethanol yield in this study. G-6-P: glucose; F-6-P: fructose-6-phosphate; F-1,6-BP: Fructose 1,6-bisphosphate; DHAP: dihydroxyacetone phosphate; GL3P: glycerol-3-phosphate; DHA: dihydroxyacetone; G-3-P: glyceraldehyde-3-phosphate; PEP: phosphoenolpyruvic acid; PYR: pyruvic acid; α-KET: α-ketoglutarate; Suc: Succinic acid; OAA: Oxaloacetic acid

To improve the ethanol yield and decrease the formation of by-products, Clustered Regularly Interspaced Short Palindromic Repeats Cas9 (CRISPR-Cas9) approach was used to knock out *S. cerevisiae GPD2*, *FPS1*, *ADH2*, and *DLD3* to modify the metabolic pathway in this study (Fig. 1). The fermentation characteristics of the engineered *S. cerevisiae* strain were investigated to analyze the effect of gene deletion on the production of ethanol and by-products. In addition, the molecular mechanism was comprehensively elucidated based on the metabolic flux and transcriptomics approaches.

## Results

### Effect of gene deletion on the grow of engineered *S. cerevisiae* SCGFAD

**Fig. 2:**
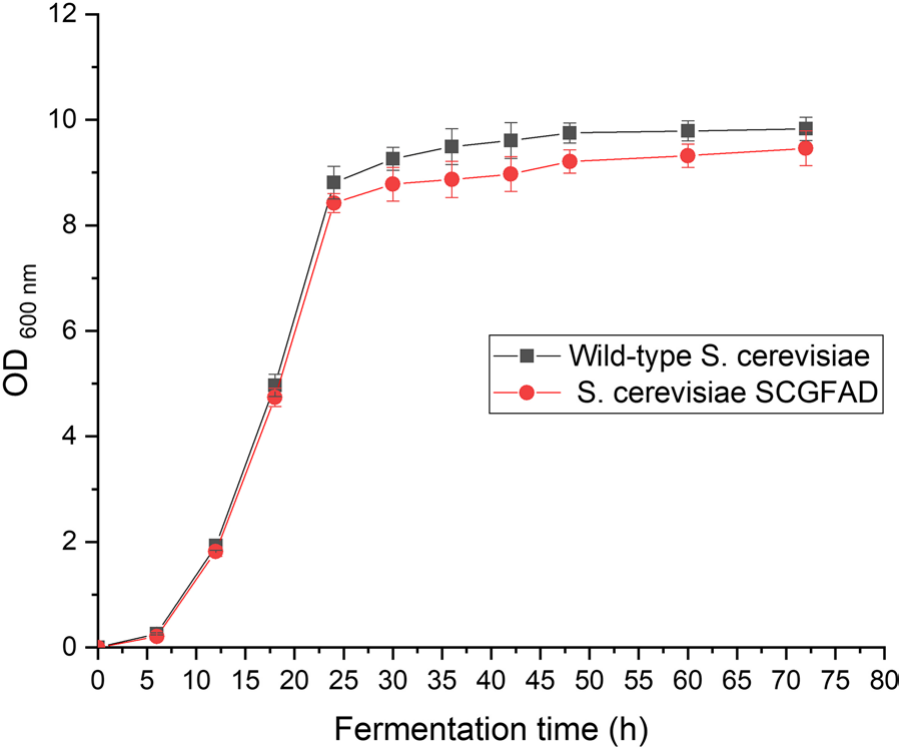
Effect of gene deletion on the growth of engineered *S. cerevisiae* SCGFAD

Four genes of *GPD2*, *FPS1*, *ADH2*, and *DLD3* in *S. cerevisiae* were successively knocked out by the CRISPR-Cas9 approach. Each deletion of the gene was confirmed by PCR and sequencing identification. The *S. cerevisiae* mutant with *GPD2*, *FPS1*, *ADH2*, and *DLD3* deletion was named *S. cerevisiae* SCGFAD. *S. cerevisiae* was inoculated into YPD liquid medium containing 50 g/L glucose. The growth of *S. cerevisiae* was determined by measuring the absorbance at the wavelength of 600 nm (Fig. 2). The result showed that *S. cerevisiae* SCGFAD possessed a similar growth curve to the wild-type strain. During the fermentation of 0–24 h, the growth curve of *S. cerevisiae* SCGFAD mutant was consistent with that of the wild-type strain. During fermentation of 24–72 h, the absorbance of *S. cerevisiae* SCGFAD at the wavelength of 600 nm was slightly below that of the wild-type strain. The OD _600 nm_ values of engineered *S. cerevisiae* SCGFAD and wild-type strain were 9.45 and 9.83 after fermentation for 72 h, respectively. Thus, the deletion of four genes of *GPD2*, *FPS1*, *ADH2*, and *DLD3* did not markedly affect the growth of engineered *S. cerevisiae* SCGFAD.

### Ethanol production and glucose consumption of *S. cerevisiae* SCGFAD

**Fig. 3:**
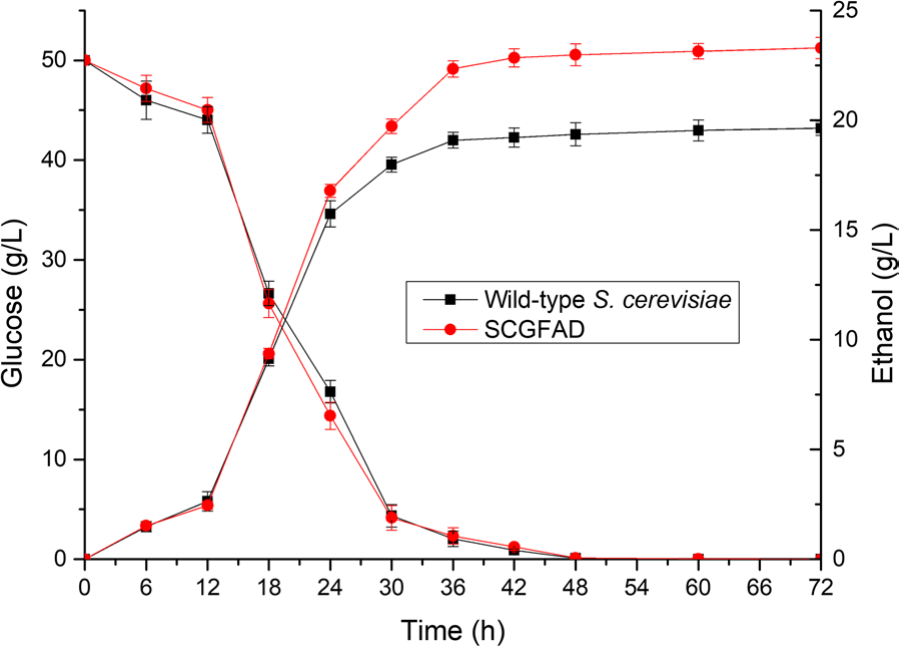
Ethanol production and glucose consumption of the wild-type strain and *S. cerevisiae* SCGFAD during fermentation

The ethanol production and glucose consumption in *S. cerevisiae* SCGFAD during fermentation were investigated in comparison with the wild-type strain. The results showed that the residual content of glucose from *S. cerevisiae* SCGFAD was similar to that from the wild-type strain. The initial glucose content of 50 g/L decreased to 0 after fermentation for 48 h (Fig. 3). During the process of glucose consumption, the ethanol content of *S. cerevisiae* SCGFAD increased simultaneously. During fermentation of 0–24 h, both *S. cerevisiae* SCGFAD and wild-type strain exhibited a similar ethanol production trend. However, during fermentation of 24–72 h, the ethanol content from *S. cerevisiae* SCGFAD was higher than that from the wild-type strain. *S. cerevisiae* SCGFAD possessed an ethanol content of 23.29 g/L, which was 1.19 folds of the wild-type strain (19.64 g/L). Thus, *S. cerevisiae* SCGFAD with *GPD2*, *FPS1*, *ADH2*, and *DLD3* deletion exhibited a higher ethanol production rate compared with the wild-type strain by modifying the metabolic pathway.

### Production of glycerol, acetic acid, and lactic acid in *S. cerevisiae* SCGFAD

**Fig. 4:**
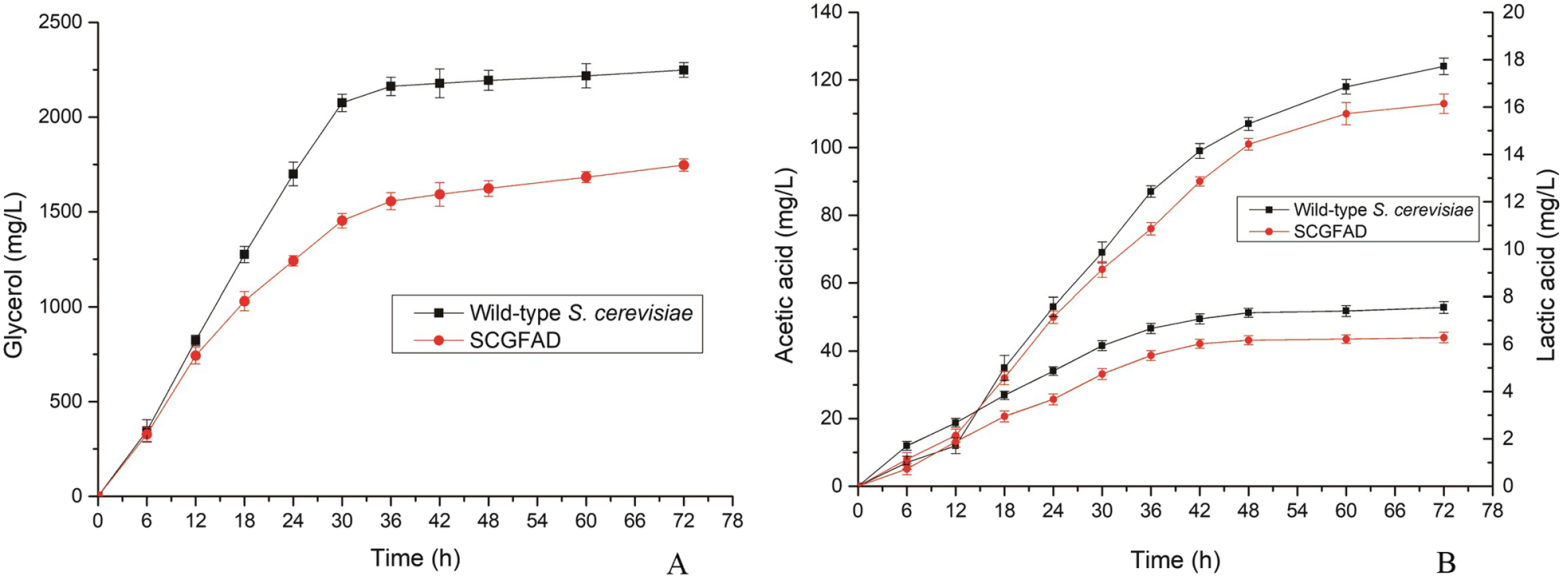
Production of glycerol, acetic acid, and lactic acid in the wild-type strain and *S. cerevisiae* SCGFAD during fermentation. **A** Glycerol content of the wild-type strain and *S. cerevisiae* SCGFAD; **B** the contents of acetic acid, and lactic acid of the wild-type strain and *S. cerevisiae* SCGFAD

Glycerol, acetic acid, and lactic acid were the by-products of *S. cerevisiae* during the fermentation for ethanol production. In this study, the contents of glycerol, acetic acid, and lactic acid during fermentation were determined to investigate the effect of gene deletion on the by-product formation (Fig. 4). During fermentation, the glycerol content in the cultivation medium from *S. cerevisiae* SCGFAD was lower than that of wild-type *S. cerevisiae* (Fig. 4A). The content of glycerol in broth increased slowly during fermentation of 0–72 h. After fermentation for 72 h, the glycerol content from *S. cerevisiae* SCGFAD was 1747 mg/L, which was 0.78-fold compared with that from the wild-type *S. cerevisiae* (2249 mg/L).

The effect of gene deletion in *S. cerevisiae* SCGFAD on acetic acid and lactic acid content was also investigated compared with the wild-type strain (Fig. 4B). The contents of acetic acid and lactic acid produced by *S. cerevisiae* SCGFAD were lower than that of wild-type *S. cerevisiae*. After fermentation of 72 h, the content of acetic acid in the fermentation broth from *S. cerevisiae* SCGFAD was 113 mg/L, which was 8.87% lower than that of wild-type *S. cerevisiae* (124 mg/L). In addition, after fermentation for 72 h, the lactic acid content in the fermentation broth from *S. cerevisiae* SCGFAD was 6.28 mg/L, which was 16.82% lower than that of wild-type *S. cerevisiae* (7.55 mg/L). These results indicated that the deletion of the four genes of *GPD2*, *FPS1*, *ADH2*, and *DLD3* resulted in the decrease of glycerol, acetate, and lactate contents in *S. cerevisiae*.

### Determination of contents and rates of metabolites

According to the fermentation characteristics of *S. cerevisiae*, metabolites were in the logarithmic stage when the fermentation time reached 18–30 h. Three-time points of 18 h, 24 h, and 30 h were selected as sampling points to measure the concentrations of glucose, ethanol, glycerol, lactic acid, acetic acid, succinic acid, and biomass. Metabolites and their contents from the wild-type and engineered *S. cerevisiae* under three different fermentation stages were recorded in Table 1.

**Table 1:** Contents of metabolites of the wild-type strain and *S. cerevisiae* SCGFAD

Metabolites	Contents (g/L)			Rates
	18 h	24 h	30 h	
Glucose (W)	26.63800	16.79000	4.35900	0.061890
Glucose (S)	25.63100	14.37400	4.14900	0.059670
Ethanol (W)	9.13400	14.34100	19.34700	0.037730
Ethanol (S)	9.68400	14.90400	20.12300	0.037820
Glycerol (W)	1.27512	1.70016	2.12520	0.002310
Glycerol (S)	1.02953	1.24115	1.45277	0.001150
Lactic acid (W)	0.00344	0.00487	0.00594	0.000007
Lactic acid (S)	0.00255	0.00367	0.00474	0.000006
Acetic acid (W)	0.03700	0.05500	0.07300	0.000098
Acetic acid (S)	0.03200	0.05000	0.06400	0.000090
Succinic acid (W)	0.00420	0.00710	0.00970	0.000011
Succinic acid (S)	0.00510	0.00820	0.01010	0.000008
Biomass (W)	5.32990	6.55270	7.77550	0.20380
Biomass (S)	5.18500	6.37360	7.56220	0.19810

The rate units of biomass and other metabolites were g/L/h and C mol/ (L h), respectively. W and S letters represented the wild-type strain and engineered *S. cerevisiae* SCGFAD, respectively

### Stoichiometric model

According to the metabolic flux model and the chemical reaction equations of the metabolites (Additional file 1), the rate equations of the intermediate metabolites were obtained (Table 2). Total of 15 reactions were used to represent the metabolites in *S. cerevisiae*. Based on the hypothesis principle of no change in the composition of *S. cerevisiae* cells during fermentation, the demand coefficients of cell precursor in a unit of mmol/g DCW (dry cell weight) were *r*_m1_ = 2.515 *r*_m_, *r*_m2_ = 0.606 *r*_m_, *r*_m3_ = 0.601 *r*_m_, *r*_m4_ = 0.007 *r*_m_, *r*_m5_ = 0.528 *r*_m_, *r*_m6_ = 1.756 *r*_m_, *r*_m7_ = 0.876 *r*_m_, *r*_m8_ = 1.159 *r*_m_, *r*_m9_ = 0.834 *r*_m_, *r*_mCO2_ = 2.610 *r*_m_, where r_m_ represented the increase rate of cell biomass (g/h).

**Table 2:** Intermediate metabolite rate equations in *S. cerevisiae*

Metabolites	Reaction rate equations
Glucose-6-phosphate	r_s_ − r_1_ − r_2_ − r_m1_ = 0
Fructose-6-phosphate	r_1_ + 0.67r_14_ − r_3_ = 0
Dihydroxyacetone phosphate	0.5r_3_ − r_4_ − r_5_ = 0
Glycerol-3-phosphate	r_5_ − r_Glycerol_ − r_m3_ = 0
Glyceraldehyde-3-phosphate	0.5r_3_ + r_4_ + 0.33r_14_ − r_6_ = 0
3-Phosphate-glycerate	r_6_ − r_7_ − r_m4_ = 0
Phosphoenolpyruvate	r_7_ − r_8_ − r_m5_ = 0
Pyruvate	r_8_ − r_9_ − r_10_ − r_m6_ − r_Lactic acid_ = 0
Acetaldehyde	0.67r_9_ − r_11_ − r_Ethanol_ = 0
Acetate	r_11_ − r_12_ − r_Acetic acid_ = 0
Acetyl-CoA	r_12_ − r_13_ − r_m7_ = 0
α-Ketoglutarate	0.83r_13_ − r_m8_ = 0
Oxaloacetate	1.33r_10_ − r_Succinic_ − 0.67r_13_ − r_m9_ = 0
Ribose-5-phosphate	0.83r_2_ − r_14_ − r_m2_ = 0
CO_2_	rx_CO2_ + 0.33r_9_ + 0.33r_2_ + 0.2r_14_ − 0.33r_13_ − r_15_ = 0

**Fig. 5:**
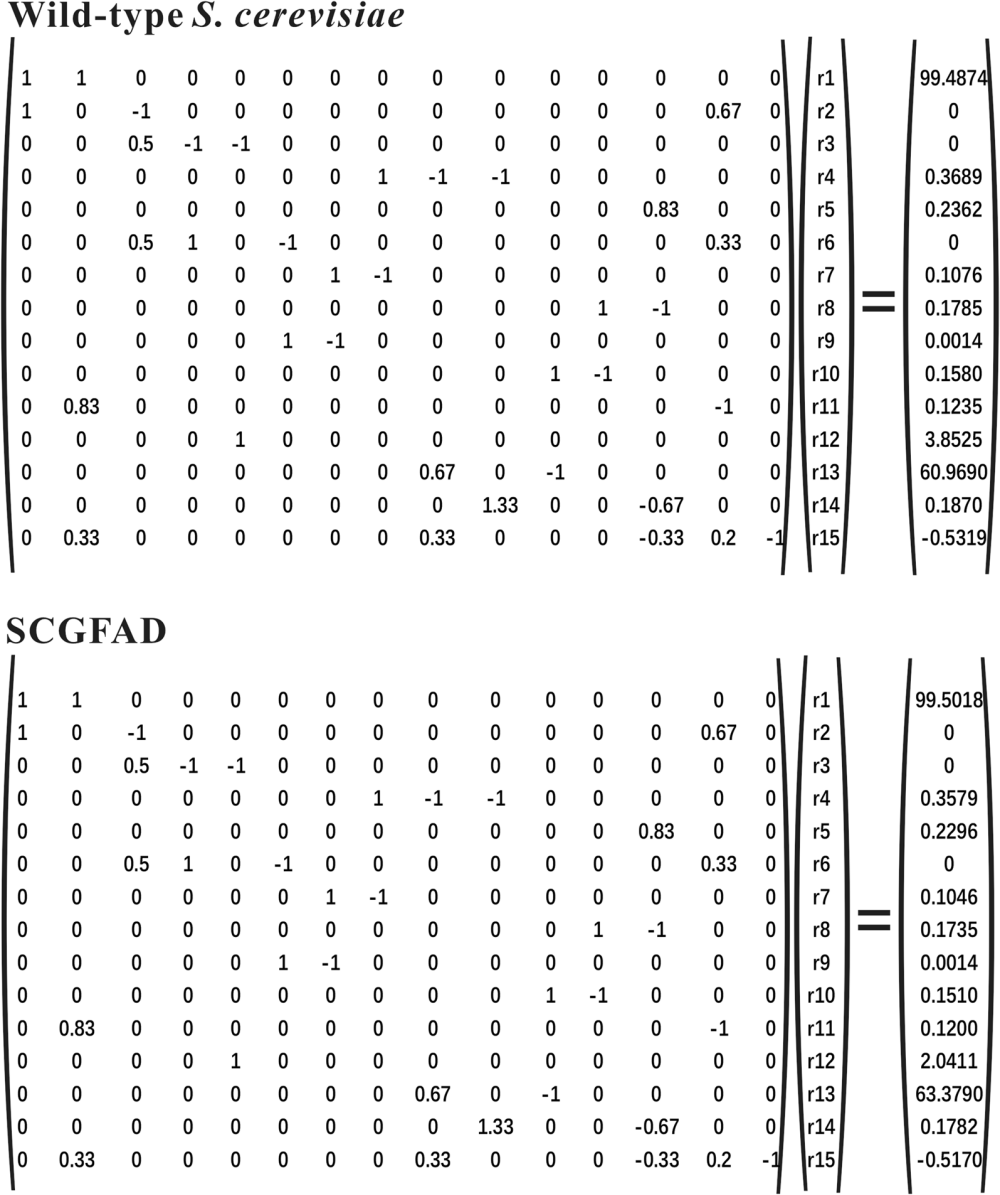
Rate equations of intermediate metabolites for the stoichiometric model analysis

The hypothesis equation for metabolic flux analysis is A × r = 0, where A represented an m × n matrix as stoichiometric coefficients, r represented an m-dimensional column vector containing metabolic reaction rates, m represented the number of intracellular reactions, and n referred to the number of intermediate metabolites. In this study, the matrix equations of wild-type *S. cerevisiae* and SCGFAD were shown in Fig. 5. On the basis of A × r = 0, the cell precursor demand coefficient and known rate were substituted into the rate equation of intermediate metabolites, and the constant term was moved to the right side of the equation.

### Metabolic flux analysis of *S. cerevisiae* SCGFAD

**Fig. 6:**
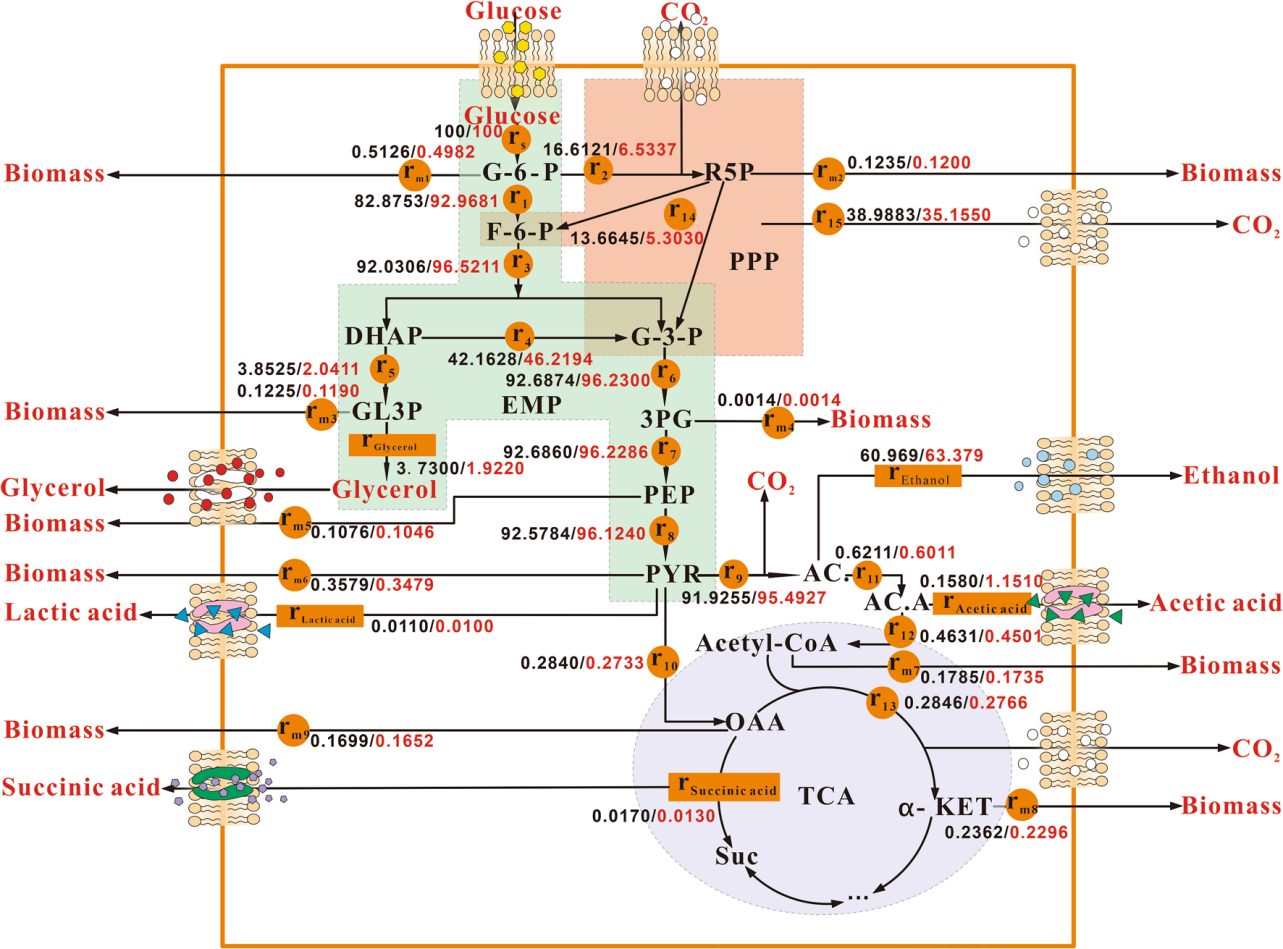
Metabolic flux distribution of *S. cerevisiae* SCGFAD. The black numbers on the left of the slash and the red numbers on the right of the slash represented the fluxes of wild-type *S. cerevisiae* and *S. cerevisiae* SCGFAD, respectively. 3PG: 3-phosphate-glycerate; AC.: acetaldehyde; AC.A: acetic acid; R5P: ribose-5-phosphate

Each flux data was normalized based on the glucose consumption rate (C·mol/ (L·h)). The metabolic flux distribution of *S. cerevisiae* SCGFAD was drawn according to the calculation results (Fig. 6). The flux r_1_(82.8753/92.9681) of the glycolytic pathway was dramatically higher than the flux r_2_(16.6121/6.5337) of the pentose phosphate pathway, which indicated that glycolysis was the main pathway of glucose carbon metabolism in *S. cerevisiae*. Metabolic flux distribution showed that deletion of *GPD2* for glycerol synthesis and *FPS1* for transport of glycerol resulted in a decrease in r_glycerol_ from 3.7300 to 1.9220. The carbon flux r_5_ for the glycerol synthesis pathway decreased dramatically from 3.8525 to 2.0411. Carbon flux redistribution resulted in carbon flux r4 for the catalysis of dihydroxyacetone phosphate to glyceraldehyde-3-phosphate increased from 42.1628 to 46.2194. The increase of carbon fluxes r6, r7, r8, and r9 for the conversion of glyceraldehyde-3-phosphate to acetaldehyde contributed to reducing glycerol production and increasing ethanol yield. In addition, *DLD3* deletion decreased the carbon flux r11 from 0.6211 to 0.6011 in the reaction node of acetaldehyde and acetate. The co-knockout of four genes increased the carbon flux r_ethanol_ from 60.969 to 63.379, which caused the increase in ethanol yield.

### *S. cerevisiae* cDNA Library and DEGs analysis

**Fig. 7:**
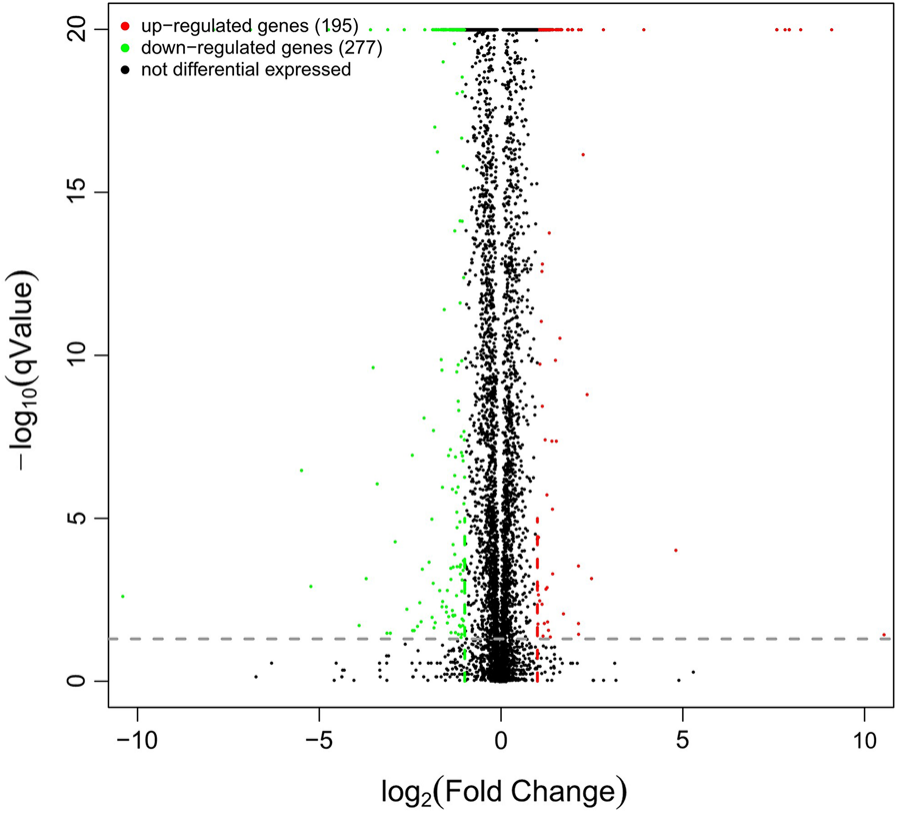
DEGs analysis of *S. cerevisiae* SCGFAD

The construction of the *S. cerevisiae* mRNA Library was performed by the following steps including accurate quantification of total RNA, mRNA purification and fragmentation, synthesis and purification of the double-stranded cDNA, end repair and dA tail addition, ligation, purification of ligation products, fragment size sorting, and library amplification. After Illumina Hiseq™ sequencing, the data were used to perform the analysis of gene expression differences. A total of 472 DEGs from *S. cerevisiae* SCGFAD were identified compared with wild-type *S. cerevisiae* (Additional file 2), in which 195 and 277 genes were significantly up-regulated and down-regulated, respectively (Fig. 7).

### GO functional annotation of differential genes

**Fig. 8:**
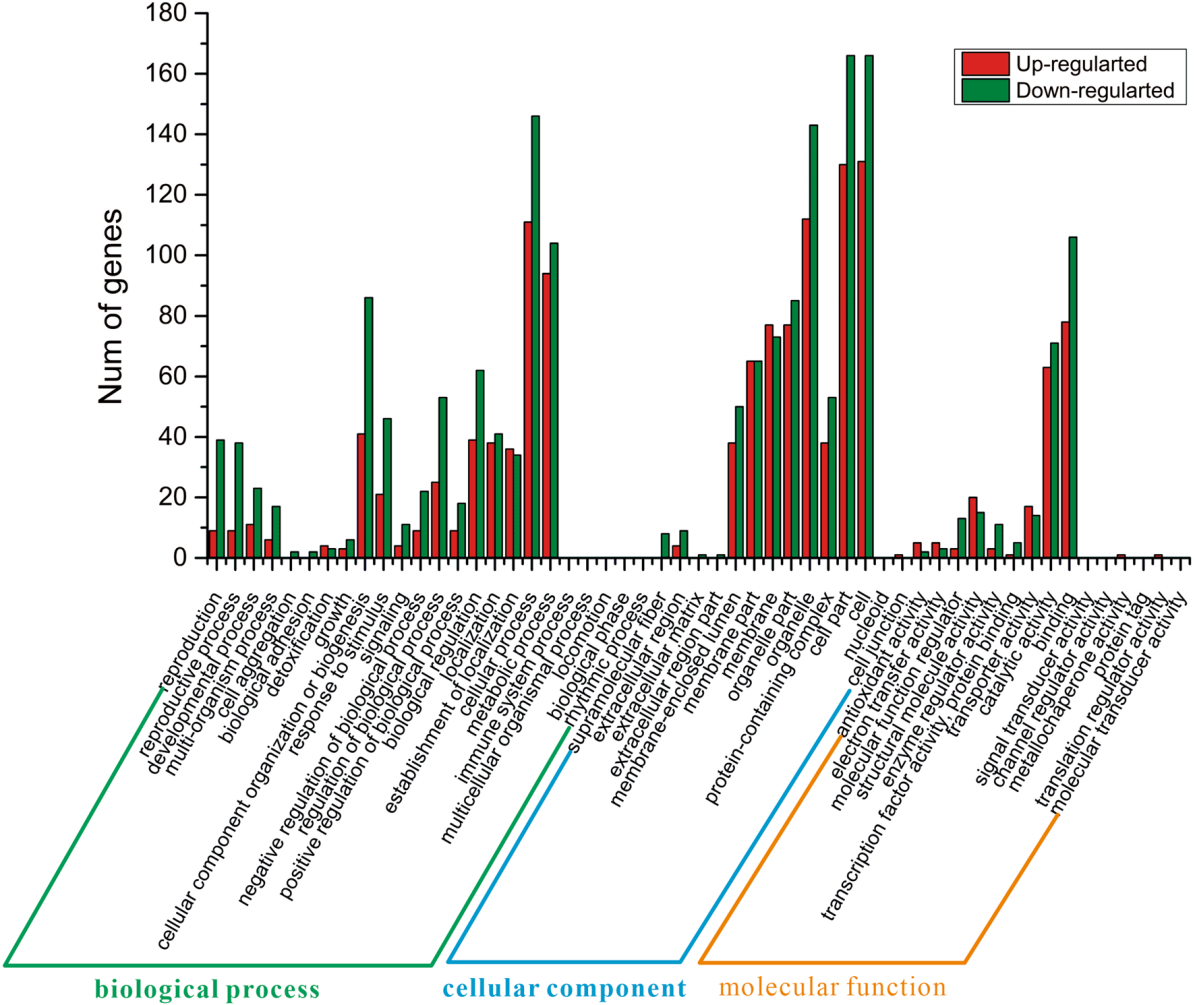
GO functional annotation of *S. cerevisiae* SCGFAD

The results of GO analysis showed that 472 DEGs were classified into three broad categories of biological processes, cellular components, and molecular function (Fig. 8). There were 19 GO terms with up- and down-regulated genes in biological processes. DEGs were mainly concentrated in the cellular component organization or biogenesis, cellular processes, and metabolic processes. There were 13 GO terms involved in the up-regulated and down-regulated genes in the cellular components. The top three categories of cells, cellular parts, and organelles accounted for the most of DEGs. In addition, 11 of GO terms were involved in the up- and down-regulated genes in the molecular function. DEGs were enriched in terms of binding and catalytic activity. In the GO analysis, the most clearly enriched term for up-regulated genes was GO:0005488 defined as binding in molecular function. The main up-regulated genes of *COX1*, *CYC7*, and *HXK1* were involved in mitochondrial electron transport and glycolysis pathways, respectively. The result indicated the gene deletion resulted in the increase of electron transfer activity and glucose decomposition in *S. cerevisiae* SCGFAD.

### KEGG enrichment analysis

**Fig. 9:**
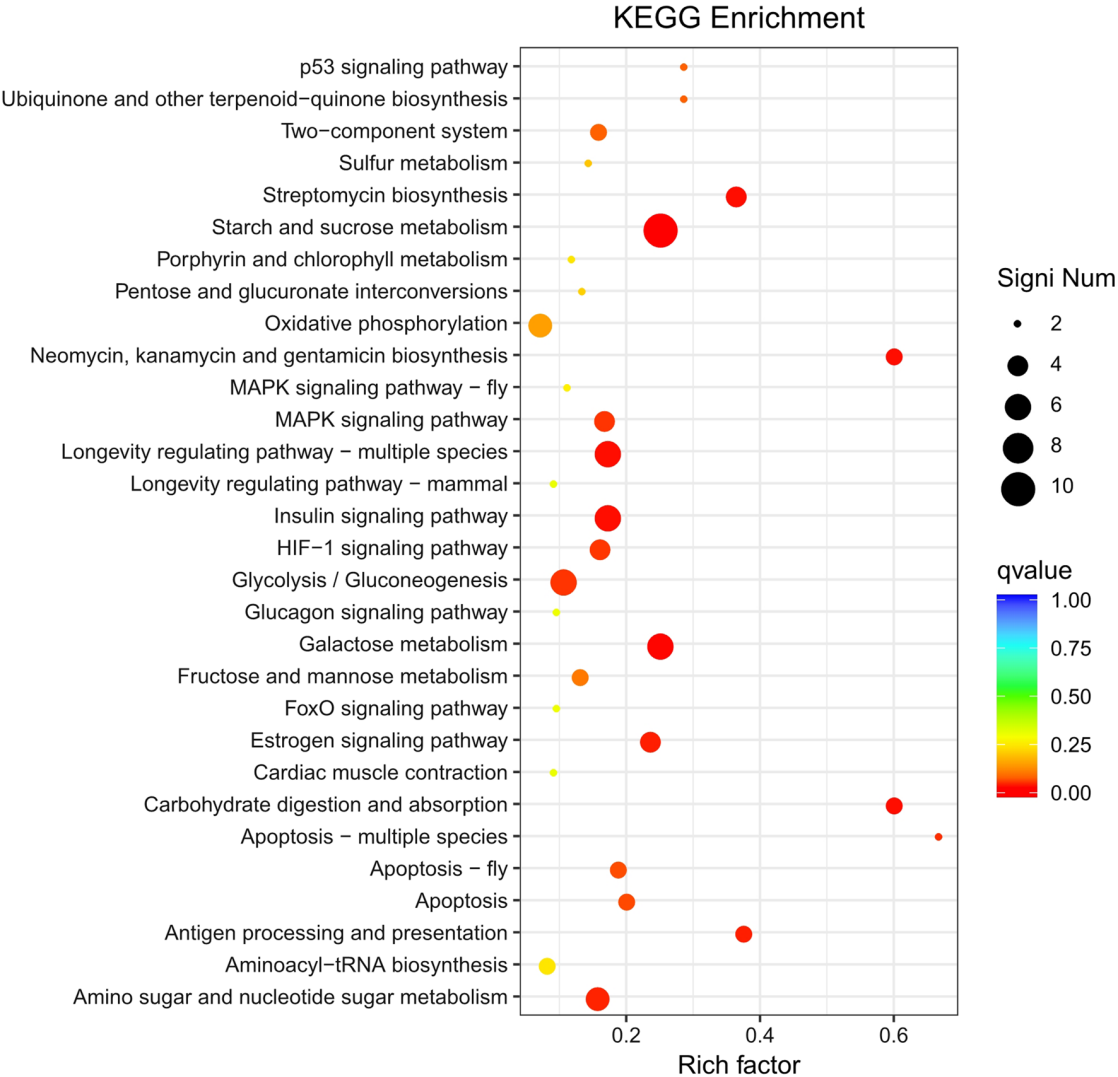
KEGG enrichment analysis for up-regulated genes

**Fig. 10:**
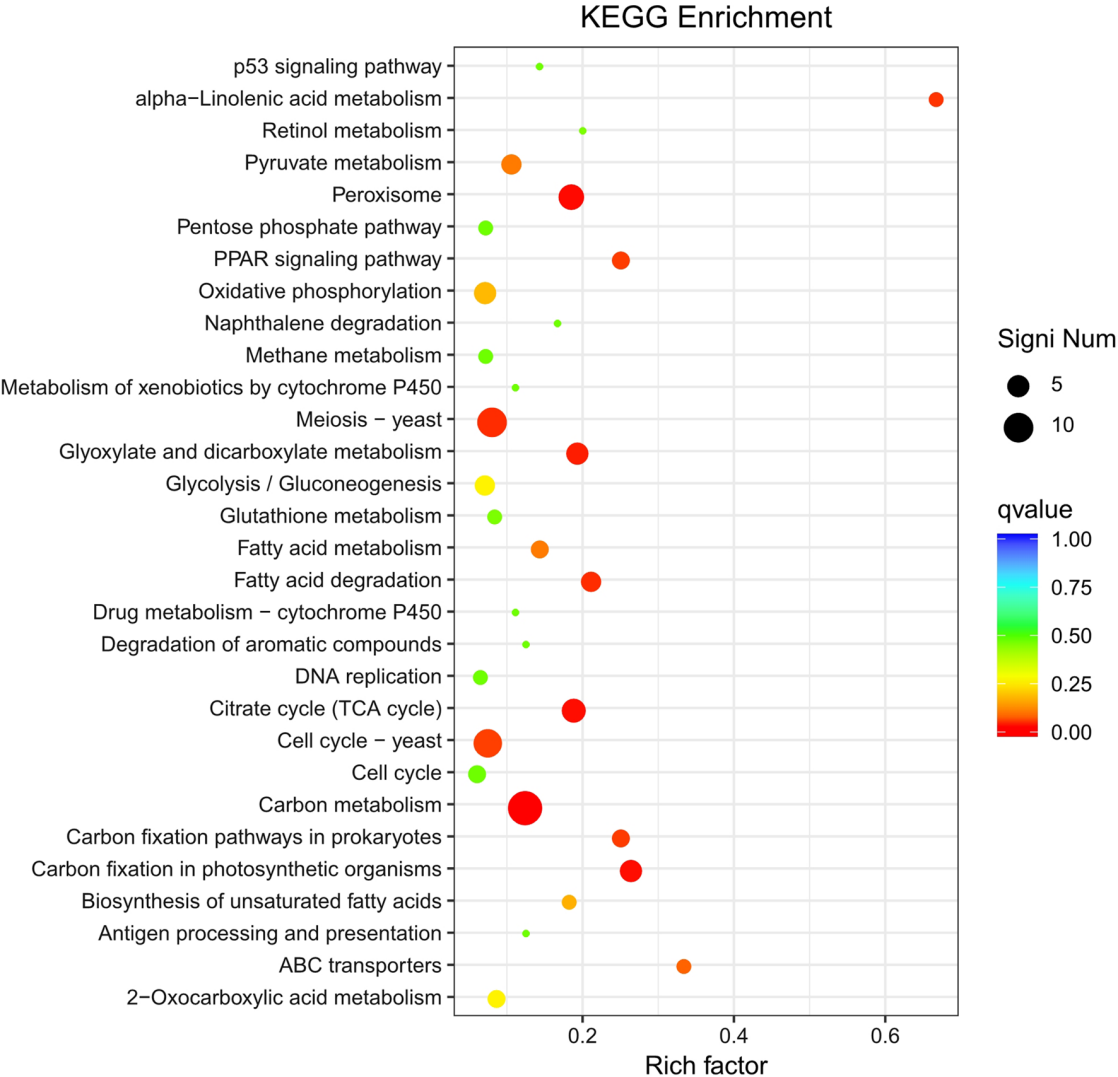
KEGG enrichment analysis for down-regulated genes

KEGG databases were used to determine whether DEGs were involved in specific pathways. The enrichment analysis of up-regulated genes included the top 30 enriched pathways among the 98 pathways (Fig. 9). The enriched pathways of up-regulated genes mainly included energy metabolism processes, such as starch and sucrose metabolism, glycolysis/gluconeogenesis, galactose metabolism, fructose, and mannose metabolism, carbohydrate digestion and absorption, and amino acid and nucleotide sugar metabolism. In addition, up-regulated genes were also enriched in signaling pathways, such as P53 signaling pathway, MAPK signaling pathway, insulin signaling pathway, and HIF-1 signaling pathway. The enrichment analysis of down-regulated genes showed only the top 30 from 79 enriched pathways (Fig. 10). The down-regulated genes were mainly enriched in acid metabolic pathways, such as pyruvate metabolism, α-linolenic acid metabolism, glyoxylate and dicarboxylic acid metabolism, and fatty acid metabolism. In addition, the meiosis-yeast and cell cycle-yeast pathways were also enriched for down-regulated genes.

## Discussion

Rational metabolic engineering was used to redirect the metabolic flow in *S. cerevisiae* by engineering metabolic pathways mainly including enzymes, transporters, and regulatory proteins based on available information [22]. *S. cerevisiae* used for bioethanol production is classified as GRAS (generally regarded as safe) by the U.S. Food and Drug Administration. However, several by-products of glycerol, yeast biomass, carbon dioxide, and other minor products during yeast fermentation lowered the total ethanol yield. The ethanol yield was increased by 12% by deleting *GPD1* and *GPD2* to completely abolish yeast’s glycerol formation. However, the growth of *S. cerevisiae* with the double mutations of *GPD1* and *GPD2* was severely affected [23]. *ADH2* plays a crucial role in the conversion of ethanol to acetaldehyde in *S. cerevisiae* [24]. The deletion of *FPS1* caused the increase of ethanol by 10% [20]. The previous reports also showed the deletion of *GPD2* and *FPS1* reduced the content of glycerol and organic acids [25, 26]. Due to multiple metabolic pathways involving the conversion of glucose to ethanol, in this study, four genes of *GPD2*, *FPS1*, *ADH2*, and *DLD3* were deleted to construct engineered *S. cerevisiae* with an increase of ethanol yield by 18.58%. In brief, this study provided a genetic modification strategy for the improvement of ethanol yield in *S. cerevisiae* by regulating the carbon flux distribution and inhibiting the by-product formation on the basis of a comprehensive analysis of ethanol metabolism.

The metabolic flux and sequencing-based RNA-Seq transcriptomics were effective methods to elaborate the molecular mechanism of arabinose fermentation in engineered *S. cerevisiae* [27]. In this study, metabolic flux analysis showed the deletion of four genes resulted in the increase of carbon flux r_ethanol_ from 60.969 to 63.379, which meant more carbon flux in ethanol production in engineered *S. cerevisiae*. KEGG enrichment analysis showed the enriched pathways of up-regulated genes were mainly involved in the energy metabolism of carbohydrates, while the down-regulated genes were mainly enriched in acid metabolic pathways. The transcriptomics analysis provided large information on gene expression and function differences in engineered *S. cerevisiae* due to gene deletion.

In this study, the lactic acid contents of the wild-type strain, *S. cerevisiae GPD2* delta *FPS1* delta *ADH2* delta mutant, and *S. cerevisiae GPD2* delta *FPS1* delta *ADH2* delta *DLD3* delta mutant were 7.55, 6.59, and 6.28 mg/L, respectively. Correspondingly, the ethanol concentrations of the above three strains were 19.64, 23.12, and 23.29 g/L, respectively. The increased amount of ethanol (0.17 g/L) was much higher than the decreased amount of lactic acid (0.31 mg/L) in *S. cerevisiae* mutant with four-gene deletion compared with three-gene deletion. Thus, *DLD3* deletion could result in the redirection of other metabolisms related to ethanol production. This study further indicated that DLD3 deletion caused the decrease of the carbon flux r11 from 0.6211 to 0.6011 in the reaction node of acetaldehyde and acetate. However, the influence degree and regulation mechanism of *DLD3* deletion on other carbon metabolism pathways need to be further clarified.

## Conclusion

Four genes of *S. cerevisiae GPD2*, *FPS1*, *ADH2*, and *DLD3* involving ethanol production were knocked out by the CRISPR-Cas9 approach. The gene deletion in engineered *S. cerevisiae* caused the increase of ethanol content by 18.58%. In addition, the contents of glycerol, acetic acid, and lactic acid decreased by 22.32, 8.87, and 16.82%, respectively. The carbon flux r_ethanol_ in engineered strain increased from 60.969 to 63.379, which represented more carbon flux in ethanol production. In addition, 472 DEGs from *S. cerevisiae* SCGFAD were identified, in which 195 and 277 genes were significantly up-regulated and down-regulated, respectively. KEGG enrichment analysis indicated the enriched pathways of up-regulated genes mainly were energy metabolism processes, and amino acid and nucleotide sugar metabolism, while the down-regulated genes were mainly enriched in acid metabolic pathways. The engineered *S. cerevisiae* strain would be applied in bioethanol industry for high-level ethanol production with less formation of by-products.

## Materials and methods

### Strains, plasmids, primers and culture conditions

The wild-type strain used for gene knockout in this study was *S. cerevisiae* S288c. *S. cerevisiae GPD2 Delta FPS1 Delta ADH2 Delta DLD3 Delta* mutant (SCGFAD) was constructed by knocking out *GPD2*, *FPS1*, *ADH2*, and *DLD3* genes of wild-type *S. cerevisiae*. Cas9-NTC and gRNA-trp-HYB were from Addgene Company (Watertown, MA, USA). *GPD2*-gRNA, *FPS1*-gRNA, *ADH2*-gRNA, and *DLD3*-gRNA plasmids carrying hygromycin B (HyB) resistance gene were amplified from gRNA-trp-HYB with the prepared primers. These plasmids expressing 20-bp gRNA were used to recognize the target gene loci of *S. cerevisiae*. The four pairs of primers were designed using the Weblink http://chopchop.cbu.uib.no/ online search system for 20 bp of gRNA sequences (Table 3). Four pairs of primers were also designed to amplify the donor DNA of *GPD2*, *FPS1*, *ADH2*, and *DLD3*. In addition, plasmid Cas9-NTC carrying nuclease and nourseothricin resistance gene was used to cut off the genomic DNA in *S. cerevisiae*. Yeast extract peptone dextrose medium (YPD) containing 20 g/L of glucose was prepared to culture *S. cerevisiae* for cell proliferation at 30 °C and 200 rpm. When the OD_600nm_ of *S. cerevisiae* reached 1, 1 mL of broth was inoculated into a 250-mL conical flask loaded with 100 mL of YPD containing 50 g/L glucose for further fermentation under the conditions of 30 °C and 200 rpm. The solid medium YPDN was prepared by the addition of 80 μg/mL NTC in YPD solid medium to screen the hypothetical transformants with Cas9-NTC integration. In addition, YPDNH medium was prepared by addition of 300 μg/mL HyB and 80 μg/mL NTC in YPD solid medium to screen the hypothetical transformants with the co-integration of Cas9-NTC and gRNA plasmid.

**Table 3:** Primers for gRNA vector construction and donor DNA used in this study

Primers	Sequence	Description
GPD2-gRNA-F1	TGATTGGTTCTGGTAACTGGGGGGTTTTAGAGCTAGAAATAGCAAG	GPD2-gRNA vector
GPD2-gRNA-R1	CCCCCAGTTACCAGAACCAATCAGATCATTTATCTTTCACTGCGGA	
Fps1-gRNA-F1	AATAAGCAGTCATCCGACGAAGGGTTTTAGAGCTAGAAATAGCAAG	FPS1-gRNA vector
Fps1- gRNA -R1	CCTTCGTCGGATGACTGCTTATTGATCATTTATCTTTCACTGCGGA	
ADH2-gRNA-F1	GGAAACATTGATGATACCGTGGGGTTTTAGAGCTAGAAATAGCAAG	ADH2-gRNA vector
ADH2-gRNA-R1	CCCACGGTATCATCAATGTTTCCGATCATTTATCTTTCACTGCGGA	
DLD3-gRNA-F1	TTGGCAGTAGTACCACAAGGTGGGTTTTAGAGCTAGAAATAGCAAG	DLD3-gRNA vector
DLD3-gRNA-R1	CCACCTTGTGGTACTACTGCCAAGATCATTTATCTTTCACTGCGGA	
Us-TV-AFB1D	5'- ATGGCTCGCGCGAAGTACTC -3'	*GPD2*
Ds-TV-AFB1D	5'-TTAAAGCTTCCGCTCTATGAA -3'	
Us-OM-PLA1	5'-TATGCGCATTTTGTCAGGGA-3'	*FPS1*
Ds-OM-PLA1	5'-GATTACATAATATCGTTCAGC-3′	
Us-DPE	5′-CAGAAAAGCGAAAGAGACACC-3′	*ADH2*
Ds-DPE	5′-TGAGGATATTATCGCAAATC-3′	
Us-AOX1	5′-GATCTAACATCCAAAGACGA-3′	*DLD3*
Ds-AOX1	5′-TCTCACTTAATCTTCTGTAC-3′	

The underlined bases were designed to recognize the target sequence using 20-bp size of RNA. The other bases were used to amplify the backbone sequences of gRNA-trp-HYB vector. The last four pairs of primers were used to amplify the donor DNA of *GPD2*, *FPS1*, *ADH2*, and *DLD3*

### Construction of SCGFAD mutant strain by CRISPR-Cas9 technology

**Fig. 11:**
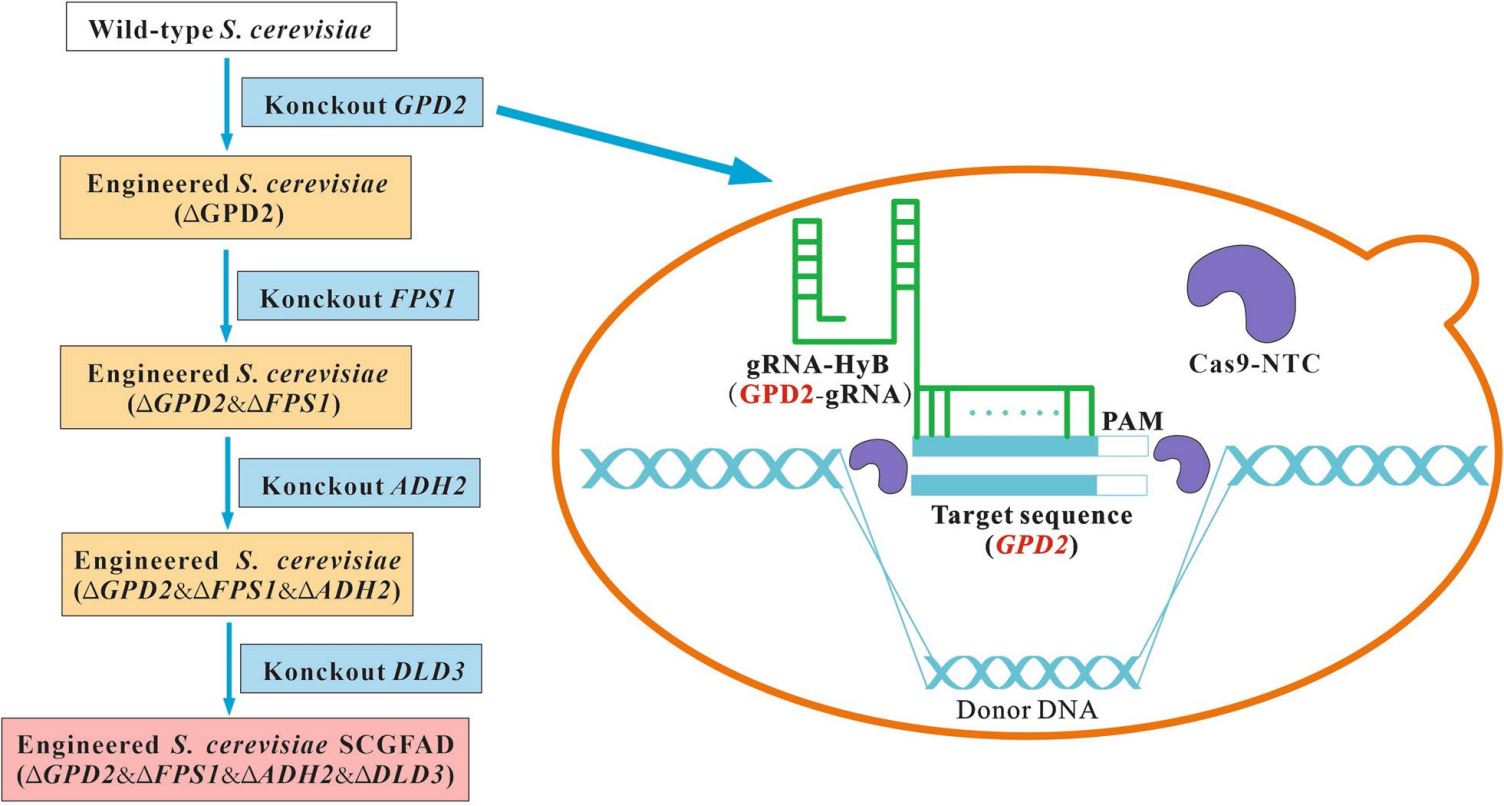
Pathway of *S. cerevisiae GPD2 Delta FPS1 Delta ADH2 Delta DLD3 Delta* mutant construction by th*e* CRISPR-Cas9 approach

*Saccharomyces cerevisiae GPD2*, *FPS1*, *ADH2*, and *DLD3* were sequentially knocked out by the CRISPR-Cas9 technology. The genetically engineered *S. cerevisiae* with *GPD2*, *FPS1*, *ADH2*, and *DLD3* deletion (SCGFAD) was constructed (Fig. 11). The transformation of plasmid in the genome of *S. cerevisiae* was carried out according to the PEG-mediated LiA-ssDNA method [28]. During the integration of *GPD2*, Cas9-NTC was transformed into the wild-type *S. cerevisiae* on the YPDN screening medium at 30 °C. The putative transformants grew on the solid screening medium containing antibiotics after an incubation of 48 h at 30 °C. Then *GPD2*-gRNA and donor DNA were transformed into the above transformants, and screened on the YPDNH solid medium. After culture for 48 h at 30 °C, the putative colonies were confirmed by PCR to amplify the donor DNA. After sequencing identification, the donor DNA was confirmed to be inserted into the genome DNA. The engineered *S. cerevisiae* was further used for the other gene integration after the loss of integrated plasmids on the antibiotic-free YPD medium.

### Effect of gene knocking out on the growth of *S. cerevisiae*

The effect of four-gene deletion on the growth and proliferation of SCGFAD mutant was investigated by the measurement of cell concentration. The fermentation broth of 1 mL with cell concentration of 1 OD_600nm_ was inoculated into a 250-mL conical flask containing 100 mL YPD. The fermentation was performed at 30 °C with a shaking speed of 200 rpm. Then, the broth samples of the SCGFAD mutant and wild-type strain were extracted every 6 h to measure the absorbance at 600 nm of wavelength.

### Determination of ethanol and by-products contents

High-Performance Liquid Chromatography (HPLC) method was used to measure the contents of ethanol and by-products. The parameters for the measurement of glucose, ethanol, and glycerol contents were a mobile phase of 0.01 mol/L H_2_SO_4_, a flow rate of 0.8 mL/min, column temperature of 50 °C with the instruments of Waters 1525 binary HPLC pump, Waters 2410 refractive index detector, and Shodex SH1011 chromatographic column [29]. In addition, the parameters for the content determination of organic acids, mainly including lactic acid, acetic acid, and succinic acid were detection wavelength of 210 nm, a mobile phase A of 10 mM KH_2_PO_4_, a mobile phase B of methanol, flow rate of 1.0 mL/min, column temperature of 30 °C with the instruments of Waters Alliance E2695, Waters 2489 UV detector, and Waters XSelect HSS chromatographic column [30]. The concentration of biomass in the medium was measured by the gravimetric method [31].

### Metabolic flux models and quasi-steady-state equation calculations

*Saccharomyces cerevisiae cerevisiae* metabolic network model was constructed according to the method established by the previous report [31]. The selected reaction pathways were glycolysis pathway (EMP), pentose phosphate pathway (PPP), and tricarboxylic acid cycle pathway (TCA). All pathways of the PPP reduce to a single reaction equation under the quasi-steady-state assumption. A simplified metabolic flux model of *S. cerevisiae* was constructed according to the above requirements. The cell precursor demand coefficients using unit of mmol/g DCW (cell dry weight) were referred to the previous report [32]. The corresponding stoichiometric model was constructed based on the metabolic flux model and reaction equation of each substance. It was assumed that the reaction in the *S. cerevisiae* cell was in a pseudo-steady state. The unknown intracellular reaction was calculated by the measured metabolite consumption or production rates. The stoichiometric model was calculated based on the carbon balance of the compound with the rate unit of C·mol/ (L·h). All the calculations were performed by Matlab Software [33].

### Construction of cDNA library

Construction of the cDNA library was performed via the following steps: (1) RNA extraction, mRNA purification, and double-stranded cDNA synthesis. The double-stranded cDNA was synthesized after mRNA was purified from total RNA. The purified cDNA samples were subjected to end A tail addition and linker ligation reactions [34]; (2) the products from the amplified cDNA library were purified by Hieff NGS™ DNA Selection Beads with 1:1 of Beads and DNA; (3) the cDNA library was sequenced by Illumina Hiseq™ [35].

### Transcriptome analysis

The original image data file processed by Illumina Hiseq™ was analyzed by CASAVA (Base Calling). After the conversion into the original sequencing sequence, Trimmomatic was used to remove sequences with N bases, linker sequences in reads, low-quality sequences (*q* < 20), and then the clean data were obtained [36, 37]. The obtained sequences after quality control were compared with the reference genome (S288c) by HISAT2 [38]. The results were counted by the RSeQC method [39]. According to the sequencing results, Transcripts Per Million (TPM) were used to estimate the sample expression. Differential expression gene (DEG) sequence was used for differential analysis, and Gene Ontology (GO), Kyoto Encyclopedia of Genes and Genomes (KEGG) were used to annotate and analyze the functions of differential genes [40, 41]. Genes with *q* value < 0.5 and fold differences |FoldChange|> 2 were defined as significantly different genes.

## Supplementary Information

**Additional file 1. **Reaction equations of metabolites in *S. cerevisiae.*

**Additional file 2. ***S. cerevisiae* DEGs.

## Data Availability

All the data generated in the study are included in the present manuscript. In addition, the reaction equations and DEGs have been listed in Additional files.
