# Supplementary material for: Ethanol yield improvement in Saccharomyces cerevisiae GPD2 Delta FPS1 Delta ADH2 Delta DLD3 Delta mutant and molecular mechanism exploration based on the metabolic flux and transcriptomics approaches
Source: Microb Cell Fact. 2022 Aug 13;21:160. doi: 10.1186/s12934-022-01885-3 (PMC9375381; doi:10.1186/s12934-022-01885-3)
Supplement: Supplementary file 1 — Additional file 1. Reaction equations of metabolites in S. cerevisiae. [file 12934_2022_1885_MOESM1_ESM.docx]

**Supplementary file 1** Reaction equations of metabolites in *S. cerevisiae*

| Reaction equations |
| --- |
| Glucose + 1/6 ATP → Glucose-6-phosphate + 1/6 ADP + 1/6 H^+^ |
| Glucose-6-phosphate → Frucose-6-phosphate |
| Frucose-6-phosphate + 1/6 ATP→1/2 Dihydroxyacetone-phosphate + 1/2 Glyceraldehyde-3-phosphate + 1/6 ADP + 1/6 H^+^ |
| Dihydroxyacetone-phosphate + 1/3 NADH + 1/3 H^+^ → Glycerol-3-phosphate + 1/3 NAD^+^ |
| Glycerol-3-phosphate + 1/3 H^+^ → Glycerol + 1/3 Pi |
| Glyceraldehyde-3-phosphate + 1/3 NAD^+^ + 1/3 ADP + 1/3 Pi → 3-phosphate-glycerate + 1/3 ATP + 1/3 NADH + 1/3 H^+^ |
| 3-phosphate-glycerate → Phosphoenolpyruvate + 1/3 H_2_O |
| Phosphoenolpyruvate + 1/3 ADP + 1/3 H^+^ → Pyruvate + 1/3 ATP |
| Pyruvate + CoA + NAD^+^→Acetyl-CoA+NADH+CO_2_ |
| Pyruvate + NADH+ H^+^→Lactic acid + NAD^+^+2H |
| Pyruvate + 1/3 H^+^→ 2/3 Acetaldehyde + 1/3 CO_2_ |
| Acetaldehyde + 1/2 NADH + 1/2 H^+^ → Ethanol + 1/2 NAD^+^ |
| Acetaldehyde + 1/2 NADP+ + 1/2 H_2_O → Acetate + 1/2 NADPH + 1/2 H^+^ |
| Acetate + 1/2 CoA + ATP → Acetyl-CoA + ADP + Pi |
| 1/2 Acetyl-CoA + 1/2 Oxaloacetate + 1/6 H_2_O + 1/3 NADP^+^ → 5/6 α-Ketoglutarate + 1/6 CO_2_ + 1/3 CoA + 1/3 NADPH + 1/6H^+^ |
| Oxaloacetate + 1/2 NADH + 1/2 H^+^→ Succinate + 1/2 NAD^+^ + 1/2 H_2_O |
| 3/4 Pyruvate + 1/4 CO_2_ + 1/4 H_2_O + 1/4 ATP → Oxaloacetate + 1/4 ADP + 1/4 Pi + 1/4 H^+^ |
| Glucose-6-phosphate + 1/3NADP+ + 1/2H_2_O →5/6 Ribose-5-phosphate + 1/3 NADPH + 1/3 H^+^ + 1/6 CO_2_ |
| Ribose-5-phosphate→ 2/5 Erythrose-4-P + 3/5 Rrucose-6-phosphate |
| Ribose-5-phosphate + 2/5 Erythrose-4-phosphate → 6/9 Frucose-6-phosphate + 3/9 Glyceraldehyde-3-phosphate |
