# Supplementary material for: Ethanol yield improvement in Saccharomyces cerevisiae GPD2 Delta FPS1 Delta ADH2 Delta DLD3 Delta mutant and molecular mechanism exploration based on the metabolic flux and transcriptomics approaches
Source: Microb Cell Fact. 2022 Aug 13;21:160. doi: 10.1186/s12934-022-01885-3 (PMC9375381; doi:10.1186/s12934-022-01885-3)
Supplement: Supplementary file 2 — Additional file 2. S. cerevisiae DEGs. [file 12934_2022_1885_MOESM2_ESM.docx]

Supplementary file 2 *S. cerevisiae* DEGs

| Gene id | MeanTPM (SC7) | MeanTPM (SC4) | Log2FoldChange | *p*Value | *q*Value | Result |
| --- | --- | --- | --- | --- | --- | --- |
| YCR010C | 6510.667 | 19423.14 | -1.5769 | 0 | 0 | down |
| YMR303C | 7658.98 | 17770.53 | -1.21426 | 0 | 0 | down |
| YFR053C | 5229.175 | 1450.381 | 1.850151 | 0 | 0 | up |
| YAL005C | 4121.075 | 1749.063 | 1.236439 | 0 | 0 | up |
| YAL054C | 1365.682 | 3349.431 | -1.29429 | 0 | 0 | down |
| YDR343C | 1899.573 | 490.8635 | 1.952282 | 0 | 0 | up |
| YDR342C | 1735.839 | 444.3868 | 1.965745 | 0 | 0 | up |
| YKR097W | 1008.524 | 2792.113 | -1.46911 | 0 | 0 | down |
| YIL057C | 4461.042 | 11002.03 | -1.30232 | 0 | 0 | down |
| YBR067C | 4670.199 | 10172.65 | -1.12314 | 0 | 0 | down |
| YDR256C | 771.0508 | 2077.868 | -1.43021 | 0 | 0 | down |
| YLL024C | 1529.184 | 629.5964 | 1.280263 | 0 | 0 | up |
| YLR377C | 398.8163 | 1459.549 | -1.87173 | 0 | 0 | down |
| YER067W | 2850.684 | 896.9434 | 1.668219 | 0 | 0 | up |
| YER065C | 752.6223 | 1648.728 | -1.13136 | 0 | 0 | down |
| YNL117W | 619.6053 | 1414.651 | -1.19103 | 0 | 0 | down |
| YPR160W | 580.7304 | 220.1438 | 1.399422 | 0 | 0 | up |
| YLR174W | 399.5727 | 1139.626 | -1.51203 | 0 | 0 | down |
| YKR009C | 243.4041 | 621.259 | -1.35184 | 0 | 0 | down |
| YCR021C | 1529.234 | 614.9897 | 1.314175 | 0 | 0 | up |
| YGL037C | 2763.024 | 1258.649 | 1.134372 | 0 | 0 | up |
| YGR236C | 2388.782 | 5587.799 | -1.226 | 0 | 0 | down |
| RDN37-1 | 2.384289 | 64.18813 | -4.75068 | 0 | 0 | down |
| YBR126W-A | 2316.323 | 329.6103 | 2.813003 | 0 | 0 | up |
| YHL021C | 724.1802 | 273.5268 | 1.404667 | 0 | 0 | up |
| YKL035W | 846.2363 | 373.8224 | 1.178707 | 0 | 0 | up |
| YMR105C | 774.0888 | 358.6526 | 1.109912 | 0 | 0 | up |
| YIL162W | 594.9487 | 234.6986 | 1.341956 | 0 | 0 | up |
| YDR150W | 66.18475 | 151.9148 | -1.19869 | 0 | 0 | down |
| YML100W | 418.5079 | 208.9424 | 1.002149 | 0 | 0 | up |
| YHR087W | 1997.484 | 706.08 | 1.50028 | 0 | 0 | up |
| YFR015C | 527.004 | 252.6995 | 1.060391 | 0 | 0 | up |
| YLR295C | 0.282423 | 1036.055 | -11.841 | 0 | 0 | down |
| YBL075C | 280.6655 | 599.1945 | -1.09417 | 0 | 0 | down |
| YEL039C | 1630.078 | 528.6896 | 1.624448 | 0 | 0 | up |
| YGR142W | 617.0618 | 263.0012 | 1.230345 | 0 | 0 | up |
| YER066C-A | 979.1471 | 310.679 | 1.656101 | 0 | 0 | up |
| YHR139C | 670.1373 | 265.1455 | 1.337672 | 0 | 0 | up |
| YHR099W | 30.49899 | 71.93411 | -1.23791 | 0 | 0 | down |
| YPR006C | 158.8247 | 402.6083 | -1.34194 | 0 | 0 | down |
| YLR177W | 275.9406 | 96.42257 | 1.516915 | 0 | 0 | up |
| YLR109W | 1450.948 | 648.4207 | 1.161994 | 0 | 0 | up |
| YJL052W | 577.5658 | 226.5056 | 1.350438 | 0 | 0 | up |
| YDR516C | 572.3851 | 284.458 | 1.00877 | 0 | 0 | up |
| YJL045W | 82.70143 | 243.252 | -1.55647 | 0 | 0 | down |
| YJR095W | 136.5689 | 431.1159 | -1.65845 | 0 | 0 | down |
| YPR184W | 164.0996 | 80.59621 | 1.025788 | 0 | 0 | up |
| YGL205W | 149.0571 | 324.9028 | -1.12414 | 0 | 0 | down |
| YER103W | 312.9045 | 139.037 | 1.170254 | 0 | 0 | up |
| YER067C-A | 1149.307 | 373.8109 | 1.620384 | 0 | 0 | up |
| YOL126C | 176.41 | 455.2226 | -1.36764 | 0 | 0 | down |
| YDR384C | 151.3358 | 467.3293 | -1.62669 | 0 | 0 | down |
| YBR126W-B | 87.95654 | 1061.473 | -3.59313 | 0 | 0 | down |
| YER015W | 131.8023 | 287.2179 | -1.12377 | 0 | 0 | down |
| YER053C | 485.4586 | 193.5525 | 1.326623 | 0 | 0 | up |
| YIL160C | 184.4853 | 428.4842 | -1.21574 | 0 | 0 | down |
| YMR169C | 213.9456 | 72.27882 | 1.565599 | 0 | 0 | up |
| YNL030W | 1410.15 | 586.6901 | 1.265178 | 0 | 0 | up |
| YHR104W | 435.4332 | 179.9219 | 1.275081 | 0 | 0 | up |
| YNR034W-A | 851.4932 | 237.0061 | 1.845071 | 0 | 0 | up |
| YGR161C-C | 82.14467 | 0.365654 | 7.811544 | 0 | 0 | up |
| YGL199C | 227.3402 | 14.97011 | 3.924696 | 0 | 0 | up |
| YBR050C | 138.2878 | 364.8794 | -1.39975 | 0 | 0 | down |
| YFL007W | 47.33496 | 96.03087 | -1.02059 | 0 | 0 | down |
| YPL147W | 75.87261 | 175.7495 | -1.21187 | 0 | 0 | down |
| YDR111C | 161.1564 | 346.6717 | -1.10511 | 0 | 0 | down |
| YKL187C | 93.25076 | 207.9616 | -1.15713 | 0 | 0 | down |
| YHR162W | 1219.25 | 593.5149 | 1.038637 | 0 | 0 | up |
| YGR161C-D | 19.19211 | 55.1545 | -1.52297 | 0 | 0 | down |
| YER159C-A | 70.81731 | 0.234254 | 8.239885 | 0 | 0 | up |
| YMR173W | 267.4033 | 113.725 | 1.233468 | 0 | 0 | up |
| YHR214C-C | 60.51958 | 0.248582 | 7.927536 | 0 | 0 | up |
| YNL098C | 448.3856 | 218.0453 | 1.040112 | 0 | 0 | up |
| YDR210C-C | 72.48254 | 0.132905 | 9.091094 | 0 | 0 | up |
| YPL016W | 27.7277 | 73.60107 | -1.4084 | 0 | 0 | down |
| YIR016W | 479.5492 | 224.7979 | 1.09305 | 0 | 0 | up |
| YFR052C-A | 584.8671 | 164.6256 | 1.82892 | 0 | 0 | up |
| YLR106C | 11.53818 | 25.8299 | -1.16263 | 0 | 0 | down |
| YNL009W | 156.236 | 331.2404 | -1.08415 | 0 | 0 | down |
| YLR414C | 462.0478 | 217.9925 | 1.083764 | 0 | 0 | up |
| YHR214C-B | 13.35842 | 40.45796 | -1.59867 | 0 | 0 | down |
| YER160C | 14.15618 | 42.09244 | -1.57213 | 0 | 0 | down |
| YNL284C-A | 53.78314 | 0.280006 | 7.585552 | 0 | 0 | up |
| YJR048W | 1046.489 | 492.1592 | 1.088359 | 0 | 0 | up |
| YJR028W | 52.88141 | 0.217683 | 7.924388 | 0 | 0 | up |
| YPL095C | 104.1319 | 26.97352 | 1.948796 | 0 | 0 | up |
| YPL247C | 210.9345 | 99.73614 | 1.080607 | 0 | 0 | up |
| YDR210C-D | 13.90521 | 39.97855 | -1.5236 | 0 | 0 | down |
| YOR120W | 314.3568 | 145.9727 | 1.106704 | 0 | 0 | up |
| YDR277C | 223.6447 | 103.8908 | 1.10614 | 0 | 0 | up |
| YBL003C | 387.8266 | 123.5567 | 1.650238 | 0 | 0 | up |
| YBL005W-B | 2.62124 | 16.63168 | -2.66561 | 0 | 0 | down |
| YBL002W | 405.0318 | 136.5247 | 1.568873 | 0 | 0 | up |
| YLR280C | 0.699525 | 167.6022 | -7.90445 | 0 | 0 | down |
| YPL201C | 60.54784 | 155.1453 | -1.35747 | 0 | 0 | down |
| YJR029W | 10.40585 | 31.1045 | -1.57973 | 0 | 0 | down |
| YNL274C | 236.9529 | 109.0138 | 1.12009 | 0 | 0 | up |
| YNL284C-B | 9.808421 | 29.71076 | -1.59889 | 0 | 0 | down |
| YOL147C | 188.2894 | 401.6618 | -1.09303 | 0 | 0 | down |
| YGL253W | 174.5717 | 82.78217 | 1.076428 | 0 | 0 | up |
| YNR014W | 211.3112 | 70.19222 | 1.589987 | 0 | 0 | up |
| YPL036W | 27.38118 | 68.42529 | -1.32135 | 0 | 0 | down |
| YKR054C | 11.8178 | 24.02082 | -1.02332 | 0 | 0 | down |
| YLR203C | 122.7239 | 47.03562 | 1.383591 | 0 | 0 | up |
| YIL111W | 338.7215 | 131.6275 | 1.363639 | 0 | 0 | up |
| YHR023W | 7.060857 | 21.8343 | -1.62868 | 0 | 0 | down |
| YPL207W | 49.52678 | 102.6645 | -1.05166 | 0 | 0 | down |
| YJR092W | 11.24749 | 31.98444 | -1.50777 | 0 | 0 | down |
| YPL141C | 29.0882 | 70.0816 | -1.2686 | 0 | 0 | down |
| YLR120C | 125.4696 | 61.57794 | 1.026852 | 0 | 0 | up |
| YNL054W-A | 30.44083 | 0.158804 | 7.582617 | 0 | 0 | up |
| YBR105C | 189.0448 | 92.49656 | 1.031257 | 0 | 0 | up |
| YLR251W | 235.3628 | 93.99292 | 1.324262 | 0 | 0 | up |
| YDR353W | 214.4543 | 105.2025 | 1.027501 | 0 | 0 | up |
| YOR385W | 150.9056 | 58.94447 | 1.356218 | 0 | 0 | up |
| YGL021W | 22.9468 | 60.56762 | -1.40025 | 0 | 0 | down |
| YLR164W | 197.4396 | 413.8531 | -1.06771 | 0 | 0 | down |
| YGL001C | 131.3808 | 54.11555 | 1.27964 | 0 | 0 | up |
| YGR067C | 37.46276 | 79.46005 | -1.08477 | 3.08E-300 | 7.66E-299 | down |
| YPL113C | 87.21317 | 177.264 | -1.02328 | 7.05E-299 | 1.74E-297 | down |
| YHR183W | 98.13207 | 43.64415 | 1.168936 | 3.22E-288 | 7.88E-287 | up |
| YNL054W-B | 6.56003 | 19.3208 | -1.55838 | 8.98E-284 | 2.17E-282 | down |
| YHR043C | 178.5824 | 76.84967 | 1.216479 | 5.50E-281 | 1.32E-279 | up |
| YJL134W | 0.212074 | 25.248 | -6.89546 | 2.80E-278 | 6.68E-277 | down |
| YPR145C-A | 250.3147 | 57.18299 | 2.130085 | 1.25E-273 | 2.95E-272 | up |
| YMR147W | 40.52884 | 127.0619 | -1.64851 | 1.71E-259 | 3.84E-258 | down |
| YIL131C | 20.99668 | 62.61636 | -1.57638 | 3.28E-259 | 7.35E-258 | down |
| YKL096W | 30.84737 | 105.2923 | -1.77118 | 1.70E-255 | 3.77E-254 | down |
| YNL073W | 74.84055 | 33.75116 | 1.148883 | 2.61E-252 | 5.72E-251 | up |
| YER032W | 8.804211 | 29.00501 | -1.72004 | 4.29E-247 | 9.21E-246 | down |
| YFR019W | 11.42997 | 23.48941 | -1.03919 | 4.05E-234 | 8.40E-233 | down |
| YGR021W | 86.31889 | 29.32145 | 1.55772 | 1.06E-230 | 2.15E-229 | up |
| YMR006C | 25.08673 | 57.58641 | -1.1988 | 3.03E-226 | 6.10E-225 | down |
| YMR084W | 163.6016 | 79.78523 | 1.035994 | 3.42E-214 | 6.75E-213 | up |
| YMR018W | 11.23789 | 39.28188 | -1.80549 | 7.46E-211 | 1.45E-209 | down |
| YPR113W | 184.0981 | 88.27226 | 1.060443 | 5.26E-210 | 1.02E-208 | up |
| YBR037C | 128.5075 | 59.69194 | 1.106244 | 7.70E-210 | 1.49E-208 | up |
| YJL063C | 157.4797 | 73.41881 | 1.100944 | 5.64E-206 | 1.08E-204 | up |
| YDR345C | 53.58734 | 22.94448 | 1.223745 | 2.55E-196 | 4.67E-195 | up |
| YML089C | 41.84949 | 149.341 | -1.83533 | 8.50E-196 | 1.55E-194 | down |
| YNL262W | 7.057629 | 16.00249 | -1.18104 | 2.27E-193 | 4.10E-192 | down |
| YNR037C | 316.4603 | 133.3652 | 1.246642 | 1.25E-192 | 2.25E-191 | up |
| YHR054W-A | 371.9084 | 140.028 | 1.409232 | 1.01E-191 | 1.79E-190 | up |
| YOR161C | 63.50895 | 29.35481 | 1.113363 | 2.72E-191 | 4.82E-190 | up |
| YMR128W | 13.8818 | 30.21998 | -1.12231 | 3.50E-191 | 6.18E-190 | down |
| YMR210W | 70.25863 | 31.27671 | 1.167587 | 7.25E-190 | 1.28E-188 | up |
| YNL270C | 19.36407 | 48.59615 | -1.32746 | 4.15E-183 | 7.16E-182 | down |
| YCL014W | 8.603358 | 19.8599 | -1.20689 | 4.23E-183 | 7.29E-182 | down |
| YJL213W | 72.70812 | 28.55526 | 1.34836 | 3.01E-180 | 5.14E-179 | up |
| YHR005C-A | 333.1232 | 153.9526 | 1.113569 | 2.17E-174 | 3.68E-173 | up |
| YLR430W | 8.709008 | 17.87102 | -1.03704 | 2.45E-174 | 4.14E-173 | down |
| YLR069C | 40.92604 | 18.96049 | 1.110023 | 1.49E-173 | 2.50E-172 | up |
| YBR056W-A | 503.5927 | 242.1794 | 1.056181 | 6.83E-173 | 1.14E-171 | up |
| YKL087C | 103.4173 | 40.90011 | 1.338301 | 8.82E-172 | 1.46E-170 | up |
| YDR072C | 55.69662 | 25.30393 | 1.138229 | 4.14E-170 | 6.73E-169 | up |
| YKL065W-A | 223.0961 | 476.7492 | -1.09557 | 1.54E-168 | 2.49E-167 | down |
| YKL050C | 16.63733 | 36.17314 | -1.1205 | 2.36E-166 | 3.77E-165 | down |
| YJL196C | 74.20651 | 29.83914 | 1.31434 | 3.57E-166 | 5.69E-165 | up |
| YDR116C | 93.12438 | 41.02504 | 1.182654 | 2.86E-163 | 4.48E-162 | up |
| YIR017C | 4.853916 | 42.19745 | -3.11993 | 1.72E-159 | 2.67E-158 | down |
| YIL144W | 26.11081 | 53.28045 | -1.02896 | 4.47E-159 | 6.92E-158 | down |
| YLR439W | 85.0902 | 38.55852 | 1.141943 | 2.59E-158 | 3.98E-157 | up |
| YMR032W | 19.78097 | 44.26322 | -1.162 | 4.13E-157 | 6.28E-156 | down |
| YNL068C | 20.63808 | 41.94359 | -1.02314 | 8.52E-155 | 1.29E-153 | down |
| YLL009C | 442.1235 | 216.499 | 1.030089 | 1.23E-152 | 1.82E-151 | up |
| YOR153W | 9.232931 | 20.09524 | -1.12199 | 7.12E-152 | 1.05E-150 | down |
| YOR180C | 55.82406 | 118.035 | -1.08026 | 1.50E-150 | 2.18E-149 | down |
| YLR090W | 64.17262 | 31.07378 | 1.04626 | 6.20E-150 | 9.01E-149 | up |
| YMR076C | 7.274667 | 18.07697 | -1.3132 | 1.55E-149 | 2.23E-148 | down |
| YJL089W | 16.56254 | 35.7192 | -1.10878 | 1.60E-145 | 2.24E-144 | down |
| YDR237W | 67.64179 | 27.25295 | 1.311502 | 3.03E-142 | 4.18E-141 | up |
| YBR045C | 12.84124 | 32.55436 | -1.34207 | 1.42E-139 | 1.94E-138 | down |
| YDL058W | 9.26554 | 18.56883 | -1.00294 | 1.56E-137 | 2.10E-136 | down |
| YER121W | 131.9692 | 268.2778 | -1.02353 | 4.37E-132 | 5.70E-131 | down |
| YBL004W | 5.854974 | 11.98254 | -1.0332 | 3.39E-130 | 4.37E-129 | down |
| YFL031C-A | 565.7845 | 238.283 | 1.247577 | 2.17E-127 | 2.74E-126 | up |
| YOL096C | 67.53568 | 30.10407 | 1.165691 | 3.00E-127 | 3.78E-126 | up |
| YLR312W-A | 74.13435 | 31.04416 | 1.25582 | 6.39E-127 | 8.03E-126 | up |
| YLR252W | 166.8069 | 65.68059 | 1.34464 | 8.89E-127 | 1.11E-125 | up |
| YLR086W | 9.988525 | 20.31783 | -1.0244 | 5.44E-124 | 6.67E-123 | down |
| YKR006C | 80.61652 | 37.09381 | 1.119897 | 6.23E-121 | 7.52E-120 | up |
| YOR195W | 10.97895 | 25.5046 | -1.21602 | 3.19E-120 | 3.83E-119 | down |
| YLR281C | 159.6245 | 79.43362 | 1.00686 | 3.75E-119 | 4.48E-118 | up |
| YJL060W | 42.6601 | 18.65365 | 1.193429 | 9.53E-119 | 1.13E-117 | up |
| YCR089W | 5.398963 | 12.58463 | -1.22091 | 4.50E-117 | 5.30E-116 | down |
| YHL003C | 57.1626 | 28.12773 | 1.023078 | 7.10E-116 | 8.27E-115 | up |
| YER179W | 22.30669 | 54.61115 | -1.29172 | 1.50E-115 | 1.73E-114 | down |
| YNL251C | 19.6462 | 41.79211 | -1.08898 | 8.41E-115 | 9.66E-114 | down |
| YMR049C | 14.36834 | 30.10158 | -1.06695 | 3.39E-112 | 3.82E-111 | down |
| YGL188C | 35.04245 | 150.2607 | -2.10029 | 1.01E-111 | 1.13E-110 | down |
| YBR275C | 5.80888 | 12.23301 | -1.07445 | 9.49E-110 | 1.04E-108 | down |
| YPL132W | 48.6286 | 19.44284 | 1.322566 | 4.56E-107 | 4.93E-106 | up |
| YEL057C | 31.69531 | 75.51671 | -1.25253 | 2.26E-106 | 2.43E-105 | down |
| YGL143C | 49.42546 | 23.77661 | 1.055711 | 6.69E-106 | 7.14E-105 | up |
| YMR144W | 12.68304 | 36.82703 | -1.53786 | 4.97E-105 | 5.29E-104 | down |
| YPR114W | 62.70877 | 29.99696 | 1.063851 | 8.36E-104 | 8.82E-103 | up |
| YJR101W | 53.11092 | 21.63177 | 1.295857 | 1.06E-100 | 1.09E-99 | up |
| YPR018W | 13.05599 | 29.78484 | -1.18987 | 3.13E-100 | 3.23E-99 | down |
| YBR282W | 91.43182 | 36.84935 | 1.311057 | 5.81E-97 | 5.81E-96 | up |
| YMR166C | 29.07164 | 10.07562 | 1.528744 | 6.60E-97 | 6.59E-96 | up |
| YFL032W | 138.5208 | 59.41584 | 1.221184 | 3.97E-96 | 3.95E-95 | up |
| YEL055C | 10.48523 | 21.44663 | -1.03239 | 4.85E-96 | 4.81E-95 | down |
| YIL158W | 45.14961 | 96.52674 | -1.09622 | 9.51E-96 | 9.42E-95 | down |
| YNL185C | 89.85918 | 38.69538 | 1.215505 | 5.28E-92 | 5.05E-91 | up |
| YFL008W | 7.848349 | 16.20409 | -1.0459 | 4.12E-89 | 3.85E-88 | down |
| YPR007C | 15.43499 | 31.03057 | -1.00749 | 7.46E-89 | 6.95E-88 | down |
| YNR041C | 35.22925 | 14.86287 | 1.245061 | 4.04E-88 | 3.72E-87 | up |
| YNL207W | 17.82782 | 39.46126 | -1.14631 | 7.94E-88 | 7.30E-87 | down |
| YKL053C-A | 175.6915 | 80.57224 | 1.12469 | 1.59E-87 | 1.45E-86 | up |
| YOR150W | 93.94592 | 43.80642 | 1.100688 | 1.90E-85 | 1.71E-84 | up |
| YBL009W | 6.234131 | 16.70879 | -1.42235 | 6.14E-84 | 5.40E-83 | down |
| YKL170W | 115.5451 | 55.5465 | 1.056688 | 1.53E-83 | 1.34E-82 | up |
| YMR024W | 36.79978 | 16.9524 | 1.118208 | 6.23E-82 | 5.34E-81 | up |
| YMR198W | 7.636084 | 19.62013 | -1.36143 | 6.16E-81 | 5.21E-80 | down |
| YPL200W | 48.81349 | 104.63 | -1.09995 | 2.67E-80 | 2.24E-79 | down |
| YGR232W | 66.8518 | 32.02882 | 1.061596 | 2.74E-80 | 2.29E-79 | up |
| YBR051W | 36.34362 | 95.53696 | -1.39436 | 4.86E-80 | 4.06E-79 | down |
| YMR157C | 51.0622 | 22.84744 | 1.160223 | 8.67E-79 | 7.13E-78 | up |
| YKR085C | 54.88135 | 22.07052 | 1.314195 | 2.42E-78 | 1.99E-77 | up |
| YDR223W | 18.98221 | 38.40264 | -1.01656 | 7.93E-77 | 6.40E-76 | down |
| YNL301C | 36.4921 | 79.97257 | -1.13192 | 1.13E-76 | 9.15E-76 | down |
| YMR117C | 22.00585 | 54.39283 | -1.30553 | 2.21E-75 | 1.76E-74 | down |
| YKL036C | 110.8657 | 53.55121 | 1.049822 | 6.20E-75 | 4.94E-74 | up |
| YIL119C | 20.94614 | 42.4698 | -1.01975 | 1.31E-74 | 1.05E-73 | down |
| YGL157W | 25.8006 | 9.563643 | 1.431772 | 2.00E-74 | 1.58E-73 | up |
| YIL019W | 21.75149 | 45.54686 | -1.06624 | 2.14E-73 | 1.68E-72 | down |
| YJL074C | 4.694685 | 10.66592 | -1.18391 | 6.57E-73 | 5.11E-72 | down |
| YBL034C | 3.508533 | 8.198936 | -1.22457 | 9.78E-73 | 7.59E-72 | down |
| YBR185C | 37.42702 | 15.5792 | 1.264459 | 7.25E-72 | 5.54E-71 | up |
| YJL096W | 79.84049 | 37.32079 | 1.097141 | 1.06E-71 | 8.11E-71 | up |
| YER078C | 26.38187 | 12.84318 | 1.038544 | 8.88E-69 | 6.55E-68 | up |
| YKL014C | 3.05742 | 6.944912 | -1.18364 | 4.66E-68 | 3.39E-67 | down |
| YGR062C | 34.32276 | 15.11187 | 1.183483 | 5.28E-68 | 3.84E-67 | up |
| YGR084C | 27.06409 | 10.81603 | 1.323208 | 6.41E-68 | 4.65E-67 | up |
| YOR087W | 18.21777 | 8.580138 | 1.086273 | 2.08E-67 | 1.50E-66 | up |
| YHR216W | 14.16943 | 4.951344 | 1.51689 | 4.58E-67 | 3.28E-66 | up |
| YBR096W | 47.57201 | 21.31515 | 1.158234 | 2.34E-66 | 1.66E-65 | up |
| YDL101C | 14.12598 | 28.93625 | -1.03453 | 7.76E-66 | 5.45E-65 | down |
| YPL027W | 10.20589 | 30.57739 | -1.58306 | 9.05E-66 | 6.35E-65 | down |
| YLR390W | 93.56166 | 41.33668 | 1.178495 | 1.85E-65 | 1.30E-64 | up |
| YIR017W-A | 46.91766 | 19.71388 | 1.250919 | 7.96E-64 | 5.47E-63 | up |
| YEL020W-A | 138.4775 | 66.72887 | 1.053269 | 1.86E-63 | 1.27E-62 | up |
| YBL097W | 6.112899 | 14.19978 | -1.21594 | 2.12E-62 | 1.44E-61 | down |
| YLR263W | 4.758533 | 11.68379 | -1.29592 | 2.80E-62 | 1.90E-61 | down |
| YHR181W | 32.84618 | 12.35632 | 1.410476 | 2.80E-61 | 1.87E-60 | up |
| YHR055C | 0.0001 | 43.27279 | -18.7231 | 5.36E-59 | 3.46E-58 | down |
| YOR095C | 16.69468 | 38.8686 | -1.21922 | 7.95E-59 | 5.12E-58 | down |
| YLR261C | 35.63207 | 7.743609 | 2.202098 | 1.66E-57 | 1.05E-56 | up |
| YBL063W | 3.858137 | 8.917591 | -1.20875 | 3.49E-57 | 2.20E-56 | down |
| YHR197W | 4.748745 | 11.62107 | -1.29112 | 4.77E-57 | 3.00E-56 | down |
| YBR156C | 7.722555 | 16.44284 | -1.09031 | 6.27E-56 | 3.87E-55 | down |
| YBL091C-A | 60.52994 | 29.23206 | 1.050098 | 1.59E-55 | 9.76E-55 | up |
| YKR080W | 19.19731 | 39.15113 | -1.02815 | 1.88E-55 | 1.15E-54 | down |
| YBL074C | 13.76392 | 30.0891 | -1.12835 | 3.55E-55 | 2.16E-54 | down |
| YEL020C-B | 130.7857 | 56.9182 | 1.200243 | 4.49E-55 | 2.73E-54 | up |
| YJR053W | 4.963373 | 13.07891 | -1.39785 | 7.06E-55 | 4.28E-54 | down |
| YNL102W | 3.713889 | 7.807137 | -1.07186 | 2.41E-54 | 1.45E-53 | down |
| YDL202W | 33.80314 | 14.73451 | 1.197958 | 4.03E-54 | 2.42E-53 | up |
| YDR179C | 22.8999 | 54.26543 | -1.24469 | 1.59E-53 | 9.51E-53 | down |
| YMR158W | 49.20715 | 20.74959 | 1.245785 | 2.92E-52 | 1.71E-51 | up |
| YER185W | 8.37854 | 22.38183 | -1.41756 | 1.11E-50 | 6.31E-50 | down |
| YIL159W | 2.919329 | 6.491187 | -1.15285 | 4.83E-48 | 2.65E-47 | down |
| YOL017W | 6.017192 | 13.0846 | -1.12071 | 5.36E-48 | 2.94E-47 | down |
| YPR078C | 7.990454 | 19.57757 | -1.29285 | 3.19E-47 | 1.72E-46 | down |
| YEL049W | 45.47744 | 91.23656 | -1.00446 | 6.82E-47 | 3.66E-46 | down |
| YDL119C | 25.34534 | 11.48619 | 1.14182 | 1.03E-46 | 5.52E-46 | up |
| YDR268W | 20.98023 | 9.857216 | 1.089778 | 1.50E-44 | 7.71E-44 | up |
| YAL064W-B | 29.52076 | 65.02981 | -1.13937 | 1.20E-43 | 6.04E-43 | down |
| YDL146W | 17.44203 | 8.666071 | 1.009118 | 1.47E-42 | 7.25E-42 | up |
| YML090W | 34.23803 | 70.83295 | -1.04882 | 3.75E-42 | 1.84E-41 | down |
| YKL119C | 36.62022 | 17.71246 | 1.047876 | 1.52E-41 | 7.40E-41 | up |
| YHR092C | 9.616627 | 3.89649 | 1.303356 | 9.53E-41 | 4.58E-40 | up |
| YGL229C | 8.639183 | 4.02559 | 1.101695 | 2.10E-40 | 1.00E-39 | up |
| YLR008C | 36.6126 | 15.95204 | 1.1986 | 4.54E-40 | 2.15E-39 | up |
| YKL194C | 11.68291 | 4.748597 | 1.298827 | 9.67E-40 | 4.56E-39 | up |
| YDR087C | 11.33323 | 25.53938 | -1.17216 | 1.19E-39 | 5.63E-39 | down |
| YOR315W | 3.260378 | 10.78032 | -1.72529 | 4.23E-38 | 1.93E-37 | down |
| YML027W | 10.7961 | 21.89381 | -1.02001 | 2.47E-37 | 1.11E-36 | down |
| YPL172C | 13.16532 | 5.93309 | 1.149887 | 3.21E-37 | 1.44E-36 | up |
| YPL209C | 2.681601 | 9.20087 | -1.77868 | 4.24E-36 | 1.86E-35 | down |
| YDR493W | 44.23943 | 19.68122 | 1.168513 | 2.56E-34 | 1.09E-33 | up |
| YNR040W | 21.08448 | 9.436335 | 1.159883 | 9.22E-34 | 3.89E-33 | up |
| YAL024C | 2.46774 | 5.079891 | -1.04161 | 1.34E-33 | 5.64E-33 | down |
| YOR186W | 21.82025 | 46.55005 | -1.09311 | 1.37E-33 | 5.75E-33 | down |
| YDR021W | 4.638281 | 11.76042 | -1.34228 | 9.36E-33 | 3.86E-32 | down |
| RUF5-2 | 25.59147 | 12.46116 | 1.038225 | 8.83E-32 | 3.58E-31 | up |
| YIL114C | 21.79713 | 10.74031 | 1.021103 | 1.71E-31 | 6.88E-31 | up |
| YGL081W | 9.168338 | 19.44564 | -1.08471 | 5.30E-31 | 2.11E-30 | down |
| YCL036W | 3.109817 | 7.804582 | -1.32749 | 1.82E-30 | 7.15E-30 | down |
| YJL160C | 11.91441 | 23.9645 | -1.00819 | 2.77E-30 | 1.08E-29 | down |
| YIL096C | 5.977987 | 14.12993 | -1.24102 | 1.47E-29 | 5.69E-29 | down |
| YGL168W | 50.15331 | 24.7294 | 1.020118 | 1.26E-28 | 4.80E-28 | up |
| YHR210C | 12.14626 | 5.261786 | 1.206888 | 1.04E-27 | 3.90E-27 | up |
| YLR063W | 4.20085 | 10.66278 | -1.34383 | 1.27E-27 | 4.74E-27 | down |
| YBL039C | 5.186948 | 10.51082 | -1.01892 | 2.60E-27 | 9.62E-27 | down |
| YGR283C | 8.882777 | 17.95338 | -1.01517 | 2.63E-27 | 9.74E-27 | down |
| YNR020C | 18.58538 | 9.125921 | 1.026126 | 2.99E-26 | 1.09E-25 | up |
| YOR177C | 5.605894 | 11.64082 | -1.05418 | 7.00E-26 | 2.52E-25 | down |
| YJL037W | 9.697881 | 21.30234 | -1.13527 | 8.09E-26 | 2.91E-25 | down |
| YNL210W | 12.64322 | 5.150372 | 1.295615 | 1.13E-25 | 4.02E-25 | up |
| YCR003W | 19.9791 | 8.645003 | 1.208553 | 8.41E-25 | 2.96E-24 | up |
| YNL320W | 10.68046 | 4.183293 | 1.352263 | 2.53E-24 | 8.81E-24 | up |
| YPL253C | 3.192747 | 6.96904 | -1.12616 | 3.30E-24 | 1.14E-23 | down |
| YLR123C | 5.052722 | 18.65135 | -1.88415 | 5.74E-24 | 1.98E-23 | down |
| YFL031W | 16.74631 | 7.808838 | 1.100664 | 1.96E-23 | 6.68E-23 | up |
| YDR014W-A | 7.410511 | 18.57465 | -1.32569 | 2.48E-21 | 8.07E-21 | down |
| YMR069W | 4.456367 | 10.88029 | -1.28778 | 8.40E-21 | 2.69E-20 | down |
| YGR225W | 1.211756 | 3.660734 | -1.59503 | 3.06E-20 | 9.66E-20 | down |
| YJL220W | 12.43663 | 26.15427 | -1.07245 | 9.11E-20 | 2.84E-19 | down |
| YIL171W-A | 12.29331 | 25.72452 | -1.06527 | 2.61E-19 | 8.03E-19 | down |
| YAR073W | 3.345084 | 7.75197 | -1.21252 | 2.95E-19 | 9.04E-19 | down |
| YGL088W | 5.309864 | 0.0001 | 15.69639 | 5.98E-19 | 1.82E-18 | up |
| tL(UAA)B2 | 0.0001 | 23.14704 | -17.8205 | 1.29E-18 | 3.91E-18 | down |
| YLR154W-C | 0.001222 | 4.833782 | -11.9497 | 2.62E-18 | 7.83E-18 | down |
| YOR313C | 1.317382 | 4.666031 | -1.82452 | 3.25E-18 | 9.70E-18 | down |
| YBR186W | 2.869275 | 6.092531 | -1.08636 | 7.16E-18 | 2.11E-17 | down |
| YNL013C | 3.763356 | 12.68419 | -1.75294 | 1.93E-17 | 5.62E-17 | down |
| YIR020C-B | 4.361623 | 0.913019 | 2.256148 | 2.35E-17 | 6.82E-17 | up |
| YOR287C | 5.65266 | 11.64494 | -1.0427 | 5.40E-17 | 1.55E-16 | down |
| YER011W | 4.845591 | 10.61174 | -1.13092 | 2.71E-15 | 7.33E-15 | down |
| YOL163W | 8.422132 | 17.7073 | -1.07209 | 2.77E-15 | 7.49E-15 | down |
| YPL192C | 6.765523 | 16.38794 | -1.27636 | 5.55E-15 | 1.48E-14 | down |
| YLR415C | 16.29691 | 6.509686 | 1.323939 | 6.45E-15 | 1.72E-14 | up |
| YCL023C | 0.0001 | 3.955163 | -15.2714 | 3.78E-14 | 9.85E-14 | down |
| YML009C-A | 19.85001 | 9.04473 | 1.13399 | 5.98E-14 | 1.55E-13 | up |
| YBR277C | 16.26066 | 7.476477 | 1.120956 | 1.02E-13 | 2.60E-13 | up |
| YGR177C | 2.511246 | 5.133543 | -1.03155 | 1.59E-13 | 4.02E-13 | down |
| YOL124C | 2.362288 | 5.167305 | -1.12923 | 9.81E-13 | 2.41E-12 | down |
| Q0045 | 0.728431 | 0.0001 | 12.83058 | 1.46E-12 | 3.57E-12 | up |
| YEL030C-A | 4.241584 | 12.54479 | -1.56441 | 1.58E-12 | 3.87E-12 | down |
| YBR012C | 13.81183 | 6.422036 | 1.104802 | 3.68E-12 | 8.88E-12 | up |
| YNL184C | 9.635269 | 3.139516 | 1.617783 | 1.23E-11 | 2.92E-11 | up |
| YHR069C-A | 2.76235 | 8.656296 | -1.64785 | 5.71E-11 | 1.32E-10 | down |
| YHR053C | 17.71621 | 6.289875 | 1.493967 | 6.00E-11 | 1.38E-10 | up |
| YOL132W | 1.990629 | 4.221331 | -1.08447 | 6.20E-11 | 1.43E-10 | down |
| YOR334W | 3.796623 | 1.812397 | 1.066818 | 7.89E-11 | 1.81E-10 | up |
| YAR061W | 2.24484 | 5.095249 | -1.18254 | 8.29E-11 | 1.90E-10 | down |
| YOR314W | 0.343541 | 3.94189 | -3.52033 | 1.01E-10 | 2.31E-10 | down |
| YNL226W | 2.450926 | 7.578947 | -1.62867 | 1.21E-10 | 2.77E-10 | down |
| YIR030C | 2.768605 | 6.450497 | -1.22025 | 1.38E-10 | 3.14E-10 | down |
| YNL235C | 3.729043 | 0.723765 | 2.365212 | 7.09E-10 | 1.56E-09 | up |
| YOL131W | 6.197066 | 14.04994 | -1.18091 | 1.14E-09 | 2.50E-09 | down |
| YOR170W | 13.53493 | 6.174586 | 1.132273 | 1.64E-09 | 3.55E-09 | up |
| YOL162W | 3.084217 | 6.924332 | -1.16677 | 2.22E-09 | 4.77E-09 | down |
| YPL021W | 0.741606 | 3.224152 | -2.12019 | 3.83E-09 | 8.15E-09 | down |
| YDR210W | 3.311042 | 0.0001 | 15.015 | 7.96E-09 | 1.67E-08 | up |
| YIL141W | 1.39972 | 5.085599 | -1.86128 | 9.42E-09 | 1.97E-08 | down |
| YEL035C | 5.096372 | 10.35574 | -1.02289 | 1.01E-08 | 2.11E-08 | down |
| tV(AAC)G2 | 28.13166 | 60.36396 | -1.10149 | 1.45E-08 | 3.01E-08 | down |
| YMR247W-A | 23.29075 | 10.0692 | 1.209808 | 1.84E-08 | 3.81E-08 | up |
| YHL046W-A | 7.52778 | 15.17561 | -1.01146 | 1.85E-08 | 3.81E-08 | down |
| YFL015C | 5.353467 | 2.034285 | 1.395952 | 2.03E-08 | 4.18E-08 | up |
| snR34 | 11.61246 | 4.053914 | 1.518286 | 2.04E-08 | 4.20E-08 | up |
| YLR329W | 1.347401 | 3.543445 | -1.39497 | 3.75E-08 | 7.61E-08 | down |
| YEL075W-A | 3.185339 | 6.793338 | -1.09267 | 4.62E-08 | 9.36E-08 | down |
| YJR005C-A | 0.834102 | 4.537695 | -2.44366 | 5.62E-08 | 1.13E-07 | down |
| YMR001C-A | 4.230639 | 11.54119 | -1.44784 | 5.77E-08 | 1.16E-07 | down |
| YDR106W | 2.400937 | 5.023147 | -1.06499 | 5.88E-08 | 1.18E-07 | down |
| YHR014W | 1.539527 | 3.678167 | -1.2565 | 6.39E-08 | 1.28E-07 | down |
| YER189W | 5.548894 | 11.46545 | -1.04702 | 8.40E-08 | 1.68E-07 | down |
| tF(GAA)M | 0.213063 | 9.580813 | -5.4908 | 1.68E-07 | 3.31E-07 | down |
| YHL046C | 3.194627 | 7.744177 | -1.27746 | 2.43E-07 | 4.75E-07 | down |
| YDR241W | 7.273718 | 14.61593 | -1.00678 | 2.75E-07 | 5.36E-07 | down |
| YLL046C | 0.098314 | 1.044219 | -3.40888 | 4.44E-07 | 8.56E-07 | down |
| YGL183C | 2.040323 | 4.643518 | -1.18642 | 5.58E-07 | 1.07E-06 | down |
| YHL045W | 1.731093 | 5.30204 | -1.61486 | 5.68E-07 | 1.09E-06 | down |
| YER172C-A | 2.53439 | 6.419245 | -1.34077 | 6.51E-07 | 1.25E-06 | down |
| tK(UUU)K | 0.0001 | 8.106031 | -16.3067 | 6.87E-07 | 1.31E-06 | down |
| YHR213W | 1.963814 | 4.629031 | -1.23705 | 8.20E-07 | 1.56E-06 | down |
| YOR203W | 6.521386 | 2.725776 | 1.258512 | 9.85E-07 | 1.86E-06 | up |
| YHR213W-A | 7.416502 | 15.09592 | -1.02535 | 1.86E-06 | 3.47E-06 | down |
| YOR277C | 5.937342 | 2.232216 | 1.411341 | 2.77E-06 | 5.12E-06 | up |
| YGL032C | 4.129478 | 9.611509 | -1.2188 | 3.43E-06 | 6.30E-06 | down |
| YDR102C | 0.966535 | 3.619269 | -1.9048 | 5.70E-06 | 1.03E-05 | down |
| YNL162W-A | 5.451053 | 11.97573 | -1.13551 | 6.94E-06 | 1.25E-05 | down |
| YHR173C | 2.955952 | 6.854791 | -1.21349 | 1.06E-05 | 1.88E-05 | down |
| YPR096C | 7.941772 | 3.893159 | 1.02852 | 2.15E-05 | 3.73E-05 | up |
| YGR273C | 0.789323 | 0.0001 | 12.9464 | 2.64E-05 | 4.56E-05 | up |
| YJR157W | 0.244516 | 1.844747 | -2.91542 | 2.97E-05 | 5.12E-05 | down |
| tS(GCU)O | 0.0001 | 5.19595 | -15.6651 | 3.03E-05 | 5.21E-05 | down |
| YLR463C | 1.314217 | 3.286131 | -1.32219 | 3.65E-05 | 6.25E-05 | down |
| YPL250W-A | 0.0001 | 1.321279 | -13.6896 | 5.27E-05 | 8.95E-05 | down |
| YML047C | 0.0001 | 0.360826 | -11.8171 | 5.27E-05 | 8.95E-05 | down |
| RDN58-1 | 2.502119 | 0.089434 | 4.806183 | 5.52E-05 | 9.36E-05 | up |
| YGR114C | 1.811311 | 4.390541 | -1.27736 | 7.40E-05 | 0.0001245 | down |
| YMR158W-B | 3.425117 | 7.035776 | -1.03856 | 0.0001174 | 0.0001945 | down |
| Q0120 | 0.116728 | 0.46087 | -1.98121 | 0.0001309 | 0.0002159 | down |
| YCR102W-A | 6.045022 | 12.11285 | -1.00272 | 0.0001427 | 0.0002348 | down |
| YDR169C-A | 6.032807 | 13.06266 | -1.11455 | 0.0001571 | 0.0002575 | down |
| YJL215C | 3.178827 | 6.378614 | -1.00475 | 0.0001706 | 0.0002793 | down |
| YCL022C | 1.291047 | 0.295544 | 2.127097 | 0.0001729 | 0.000283 | up |
| YAL034C-B | 2.051875 | 4.75184 | -1.21154 | 0.0001809 | 0.0002956 | down |
| YDR281C | 2.689011 | 5.895868 | -1.13263 | 0.0001835 | 0.0002996 | down |
| YDR048C | 1.815568 | 4.528031 | -1.31846 | 0.000187 | 0.0003052 | down |
| YDR340W | 1.673046 | 4.370703 | -1.38539 | 0.0002044 | 0.0003324 | down |
| YGR109C | 0.13386 | 0.601454 | -2.16773 | 0.0002187 | 0.0003546 | down |
| YKL096C-B | 6.486282 | 13.40071 | -1.04685 | 0.0002376 | 0.0003839 | down |
| YJL119C | 0.0001 | 0.999224 | -13.2866 | 0.000285 | 0.0004573 | down |
| YOR225W | 3.34045 | 1.251167 | 1.416768 | 0.0003088 | 0.0004935 | up |
| YIR030W-A | 2.010656 | 4.377489 | -1.12244 | 0.0003111 | 0.0004971 | down |
| YLL047W | 1.403666 | 0.250619 | 2.485632 | 0.000433 | 0.0006853 | up |
| YBR196C-A | 0.174508 | 2.286829 | -3.71198 | 0.0004356 | 0.0006891 | down |
| tH(GUG)G1 | 6.208646 | 16.17439 | -1.38136 | 0.0004607 | 0.0007275 | down |
| tC(GCA)B | 11.56472 | 24.34095 | -1.07365 | 0.0004801 | 0.0007576 | down |
| tG(GCC)F1 | 0.0001 | 4.054675 | -15.3073 | 0.0005046 | 0.0007944 | down |
| YKL097C | 0.466454 | 1.703214 | -1.86845 | 0.0006126 | 0.0009587 | down |
| YFR056C | 4.432633 | 2.211174 | 1.003351 | 0.0007363 | 0.0011442 | up |
| tD(GUC)I2 | 0.11982 | 4.509246 | -5.23395 | 0.0007715 | 0.0011945 | down |
| YBL044W | 2.908962 | 1.210827 | 1.264512 | 0.0008363 | 0.0012925 | up |
| YGL118C | 2.529824 | 1.076712 | 1.232405 | 0.0009423 | 0.0014499 | up |
| YLR222C-A | 1.498937 | 4.180462 | -1.47972 | 0.0010362 | 0.0015901 | down |
| YHR212C | 0.702336 | 2.267018 | -1.69056 | 0.0010478 | 0.001607 | down |
| YGL230C | 1.651165 | 3.499051 | -1.08348 | 0.0011013 | 0.0016871 | down |
| YFR054C | 1.495408 | 2.99448 | -1.00177 | 0.0012401 | 0.0018951 | down |
| YKL118W | 4.42028 | 2.171168 | 1.025666 | 0.0014296 | 0.0021743 | up |
| YBR109W-A | 2.289083 | 5.361402 | -1.22784 | 0.0015137 | 0.002294 | down |
| Q0070 | 0.0001 | 0.135966 | -10.409 | 0.0016039 | 0.0024264 | down |
| YAL026C-A | 2.768416 | 1.327816 | 1.060006 | 0.0022148 | 0.0033183 | up |
| YAR060C | 0.702336 | 2.142734 | -1.60922 | 0.0023227 | 0.0034769 | down |
| RUF21 | 0.135321 | 0.638621 | -2.23857 | 0.0025436 | 0.0037918 | down |
| YDR431W | 3.124987 | 1.433545 | 1.124263 | 0.0028418 | 0.0042179 | up |
| snR38 | 0.0001 | 2.305283 | -14.4927 | 0.0028808 | 0.0042698 | down |
| tL(UAG)L1 | 0.0001 | 2.881966 | -14.8148 | 0.0028808 | 0.0042698 | down |
| YOR011W-A | 1.011639 | 3.125401 | -1.62735 | 0.0034257 | 0.0050417 | down |
| YHR214C-D | 0.832034 | 2.364737 | -1.50697 | 0.0034278 | 0.0050437 | down |
| YFR034W-A | 0.187408 | 1.131672 | -2.5942 | 0.0041091 | 0.0060048 | down |
| YIL012W | 0.944873 | 2.394795 | -1.34171 | 0.0045098 | 0.0065734 | down |
| YDR183C-A | 0.843457 | 2.395603 | -1.506 | 0.0050871 | 0.0073787 | down |
| tG(GCC)J2 | 0.0001 | 3.025413 | -14.8848 | 0.0052022 | 0.0075336 | down |
| YPL121C | 0.0001 | 0.293096 | -11.5172 | 0.0052022 | 0.0075336 | down |
| snR77 | 7.66474 | 15.33512 | -1.00053 | 0.0053891 | 0.0077902 | down |
| Q0105 | 0.397828 | 0.1212 | 1.714755 | 0.0057745 | 0.0083314 | up |
| YOR255W | 0.213631 | 0.0001 | 11.06091 | 0.0062687 | 0.0090235 | up |
| YHR212W-A | 1.611462 | 3.894624 | -1.27311 | 0.0063851 | 0.0091881 | down |
| YBR200W-A | 1.941706 | 4.876222 | -1.32844 | 0.0063851 | 0.0091881 | down |
| snR87 | 2.726191 | 6.790923 | -1.31672 | 0.0067394 | 0.0096766 | down |
| YAR069C | 0.873417 | 2.375951 | -1.44376 | 0.0070595 | 0.0101127 | down |
| tQ(UUG)E1 | 1.953484 | 7.284799 | -1.89884 | 0.0074518 | 0.0106546 | down |
| YAR053W | 1.55478 | 3.277773 | -1.07601 | 0.0084078 | 0.0119663 | down |
| YOR293C-A | 0.0001 | 1.225573 | -13.5812 | 0.0094473 | 0.0133954 | down |
| snR78 | 5.905211 | 12.12436 | -1.03785 | 0.0094614 | 0.0134126 | down |
| YOL160W | 0.245786 | 1.129689 | -2.20045 | 0.0102477 | 0.0144882 | down |
| YHR028W-A | 2.036953 | 0.840582 | 1.276952 | 0.0104142 | 0.0147144 | up |
| YLR308W | 0.134083 | 0.471306 | -1.81354 | 0.0109687 | 0.0154659 | down |
| YKL102C | 0.620488 | 1.729974 | -1.47928 | 0.0112328 | 0.0158153 | down |
| YER091C-A | 3.028311 | 1.32947 | 1.187662 | 0.0116559 | 0.0163706 | up |
| snR79 | 3.601198 | 0.8242 | 2.127411 | 0.0117514 | 0.016498 | up |
| YBR298C-A | 3.837909 | 1.902273 | 1.012596 | 0.0121558 | 0.0170377 | up |
| tA(UGC)E | 0.184339 | 2.762604 | -3.9056 | 0.0135464 | 0.0188936 | down |
| tV(CAC)D | 5.411491 | 11.9872 | -1.1474 | 0.0135518 | 0.0188952 | down |
| YMR013W-A | 5.164451 | 10.74664 | -1.0572 | 0.0135518 | 0.0188952 | down |
| YKL106C-A | 0.53612 | 2.642674 | -2.30137 | 0.0144994 | 0.0201527 | down |
| YOL079W | 0.944371 | 2.047815 | -1.11666 | 0.0147702 | 0.0205123 | down |
| tX(XXX)D | 1.047047 | 3.767929 | -1.84745 | 0.0160719 | 0.0222567 | down |
| YAL037C-A | 0.0001 | 1.590162 | -13.9569 | 0.0172598 | 0.0238437 | down |
| YDR271C | 1.472985 | 0.597835 | 1.300923 | 0.0193582 | 0.0265703 | up |
| snR52 | 0.506933 | 2.660307 | -2.39173 | 0.0197114 | 0.0270143 | down |
| YER084W-A | 0.093255 | 0.504766 | -2.43636 | 0.0197114 | 0.0270143 | down |
| YLR296W | 0.668582 | 1.597751 | -1.25687 | 0.0230215 | 0.0312496 | down |
| YER038W-A | 0.529545 | 1.376455 | -1.37813 | 0.0230215 | 0.0312496 | down |
| YNL146C-A | 0.100205 | 0.883978 | -3.14106 | 0.024001 | 0.0325337 | down |
| YHL037C | 0.055944 | 0.462675 | -3.04794 | 0.024001 | 0.0325337 | down |
| YOR329W-A | 0.952419 | 0.217544 | 2.130289 | 0.0260351 | 0.0351896 | up |
| tQ(UUG)L | 4.024739 | 9.101091 | -1.17714 | 0.0261316 | 0.0353096 | down |
| snR39 | 3.429951 | 7.278012 | -1.08536 | 0.0261316 | 0.0353096 | down |
| YCL074W | 0.148148 | 0.0001 | 10.53282 | 0.0269312 | 0.0363504 | up |
| YOR300W | 1.653603 | 0.743879 | 1.152473 | 0.0297149 | 0.0398712 | up |
| YKL223W | 0.0001 | 0.395305 | -11.9488 | 0.0317363 | 0.0424623 | down |
| YBR219C | 0.0001 | 0.308983 | -11.5933 | 0.0317363 | 0.0424623 | down |
| YBL108C-A | 3.210321 | 1.25656 | 1.353238 | 0.0317885 | 0.0425197 | up |
| YOR192C-C | 1.313786 | 2.824955 | -1.1045 | 0.0328142 | 0.0438229 | down |
| snR75 | 1.741902 | 4.609172 | -1.40384 | 0.0369077 | 0.0490497 | down |
